# FgJhd2 Modulates FgMpf2 Expression via H3K4 Demethylation and Influences Sexual Development in *Fusarium graminearum*

**DOI:** 10.1111/1462-2920.70354

**Published:** 2026-06-18

**Authors:** Xiwen Liao, Liqiang Yao, Jie Wang, Yingqi Chen, Shisheng Chen, Zhaomei Qi, Yuan Chen

**Affiliations:** ^1^ State Key Laboratory of Wheat Improvement Peking University Institute of Advanced Agricultural Sciences, Shandong Laboratory of Advanced Agricultural Sciences in Weifang Weifang Shandong China; ^2^ College of Life and Environmental Sciences Hangzhou Normal University Hangzhou Zhejiang China; ^3^ College of Plant Protection Shandong Agricultural University Taian Shandong China

## Abstract

Histone methylation plays a pivotal role in the epigenetic regulation of fungal development. In this study, we characterise FgJhd2, a homologue of the histone demethylase Jhd2 from the JARID family, in the plant pathogenic fungus *Fusarium graminearum*. FgJhd2 localises in the nucleus and exhibits specific H3K4me3 demethylase activity. Deletion of *FgJHD2* impaired hyphal growth and sexual reproduction, leading to smaller perithecia, defective asci and ascospore development. Transcriptomic analysis revealed significant down‐regulation of key developmental genes, including *FgAMA1*, *AMD1* and *PUK1*, in the *Fgjhd2* mutant. Combined ChIP‐seq and RNA‐seq analyses revealed that loss of *FgJHD2* leads to increased H3K4 methylation and elevated expression of *FgMPF2*, suggesting a potential negative regulatory relationship. FgMpf2, homologous to the *Schizosaccharomyces pombe* RNA‐binding protein Mpf2, contains a Pumilio domain essential for RNA degradation. Phenotypic analyses revealed that *FgMPF2* deletion abolishes ascus formation, while its overexpression disrupts ascus maturation, resembling the defects observed in the *Fgjhd2* mutant. Intriguingly, overexpression of *FgMPF2* led to reduced *FgAMA1*, *AMD1* and *PUK1* transcript levels. Our study supports a model in which FgJhd2 modulates sexual reproduction by regulating *FgMPF2* expression, which in turn influences the transcript stability of key developmental genes in *F. graminearum*.

## Graphical abstract

The histone demethylase FgJhd2 regulates sexual reproduction in the fungal pathogen *Fusarium graminearum* via H3K4me3 demethylation. Mechanistically, FgJhd2 restricts H3K4 methylation to suppress the expression of FgMpf2, an RNA‐binding protein involved in RNA degradation. This epigenetic repression stabilises essential developmental transcripts, thereby ensuring proper ascus and ascospore development.

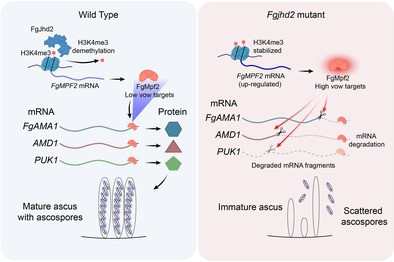

## 1 Importance

Histone methylation dynamics play pivotal roles in fungal development and virulence, yet the mechanisms linking epigenetic regulation to sexual structures morphogenesis in filamentous ascomycetes remain poorly understood. This study identifies FgJhd2 as a critical epigenetic modulator of sexual reproduction in *F. graminearum*, a major cereal pathogen. We demonstrate that FgJhd2‐mediated H3K4me3 demethylation suppresses the RNA‐binding protein FgMpf2. Overexpression of *FgMPF2* correlates with reduced transcript levels of genes essential for asci and ascospore formation (e.g., *FgAMA1*, *AMD1* and *PUK1*), suggesting a potential link between histone demethylation and RNA stability during fungal sexual development. Given the conservation of Jhd2 and Mpf2 homologues in eukaryotes, our findings shed light on evolutionarily conserved strategies for coordinating epigenetic and post‐transcriptional regulation during development. Additionally, disrupting this pathway could inform novel antifungal strategies targeting Fusarium pathogenicity.

## 2 Introduction

The homothallic pathogen *F. graminearum* is a globally distributed crop infection factor and the main pathogen of Fusarium head blight (FHB). In addition to losing yield, the infected crop is contaminated with a variety of mycotoxins produced by *F. graminearum* that can affect human and livestock health (Desjardins and Proctor 2007; Trail 2009). In the infection cycle of *F. graminearum*, sexual reproduction is particularly important. Perithecia and associated hyphae remaining on plant disease residues are regarded as the main overwintering structures of *F. graminearum* (Fernando et al. 1997). During flowering, ascospores are discharged from perithecia and are transmitted by the airflow to infect the host, which is considered to be the primary inoculum for FHB (Osborne and Stein 2007; Trail et al. 2002). Fungi rely on sexual reproduction to increase the diversity of genetic variants and eliminate deleterious mutations (Heitman et al. 2013). Perithecia production, ascospores formation and discharge play a key role in the disease cycle.

The pathways and genes that regulate sexual reproduction in *F. graminearum* have been studied, such as signal transduction pathways (Ding et al. 2023; Jenczmionka et al. 2003; Park et al. 2012), lipid metabolism pathways (Guenther et al. 2009; Wang et al. 2020), A‐to‐I RNA editing (Liu et al. 2016; Qi et al. 2024), mating genes (Zheng et al. 2013), transcription factors (Kim et al. 2025) and other key genes. Many of these genes, such as *GEA1* and *AMD1* (Cao et al. 2017), only perform specific functions in sexual reproduction, while others regulate the entire vegetative growth and development (Wang et al. 2022).

Post‐translational modification (PTM) refers to the regulation of protein activity, localisation and expression by adding or removing specific groups to protein amino acid residues. Common PTMs include phosphorylation, acetylation, methylation and ubiquitination, which regulate the growth and development of eukaryotes. Histone methylation is the type of histone post‐translational modification (HPTM) that typically occurs at the amino acid residue of the N‐terminal tail of the histone (Mosammaparast and Shi 2010). Histone methylation is a dynamically regulated process regulated by histone methyltransferases (HMTs) and histone demethyltransferases (HDMs), respectively (Ramakrishnan et al. 2016). A family of demethylases involved in the regulation of histone demethylation has been discovered, including lysine‐specific demethylases (LSDs) and demethylases containing Jumonji C (JmjC) domains (Shi et al. 2004; Tsukada et al. 2006).

Based on domain differences and sequence alignment, demethylases containing JmjC domains can be divided into seven subfamilies, namely JHDM1, JHDM2, JHDM3, JARID1, UTX/UTY, PHF8 and proteins containing only JmjC domains (Xiang et al. 2007). In mammals, members of the JARID1 subgroup have been implicated in multiple cancer types and neurological disorders (Kooistra and Helin 2012). In *Saccharomyces cerevisiae* , five histone demethylases (JHDMs) containing JmjC domains have been demonstrated, including Jhd1, Jhd2, Rph1, Gis1 and Ecm5, and have been shown to have different demethylase activities (Ryu and Ahn 2014). Jhd2, the only H3K4 demethylase in *S. cerevisiae* , belongs to the JARID family and has been shown to be associated with telomere silencing and mitotic rDNA condensation (Ryu and Ahn 2014; Huang et al. 2010; Liang et al. 2007). In *Aspergillus nidunoccus*, KdmB is a JARID1 family histone H3 lysine demethylase, homology of yeast Jhd2, which targets and demethylates H3K4me3 and mediates transcriptional downregulation and is involved in the regulation of fungal secondary metabolite synthesis (Gacek‐Matthews et al. 2016). In *Magnaporthe oryzae*, the absence of *MoJMJ1* belonging to the JARID group results in fungal vegetative growth, asexual reproduction, appressorium formation and invasive growth defects (Huh et al. 2017). In *Botrytis cinerea*, BcJar1 is an H3K4 demethylase belonging to the JARID group, which regulates H3K4me2 and H3K4me3 methylation levels during vegetative and pathogenic development, respectively. Loss of BcJar1 impairs conidiation formation, stress adaptation and infection virulence (Hou et al. 2020). Despite these advances, the role and molecular mechanisms of histone methylation modifications in fungal sexual reproduction remain largely unexplored in filamentous fungi.

In this study, we demonstrated that FgJhd2 (FGSG_05855) is an H3K4 demethylase that belongs to the ortholog of human JARID1 and yeast Jhd2. It is involved in the regulation of vegetative growth, infection and sexual reproduction of *F. graminearum*. Recent transcriptomic and functional analyses suggest that FgJhd2 may influence sexual reproduction through the regulation of FgMpf2 (FGSG_05203), a putative RNA‐binding protein homologous to the meiotic Pumilio family protein SpMpf2 in *S. pombe*. This finding raises the possibility that FgJhd2‐mediated histone demethylation fine‐tunes the expression of FgMpf2 to ensure proper meiotic differentiation and ascospore formation in *F. graminearum*.

## 3 Results

### 3.1 FgJhd2 Is a Conserved JmjC Domain‐Containing Histone Demethylase With Specific H3K4me3 Demethylation Ability

**FIGURE 1:**
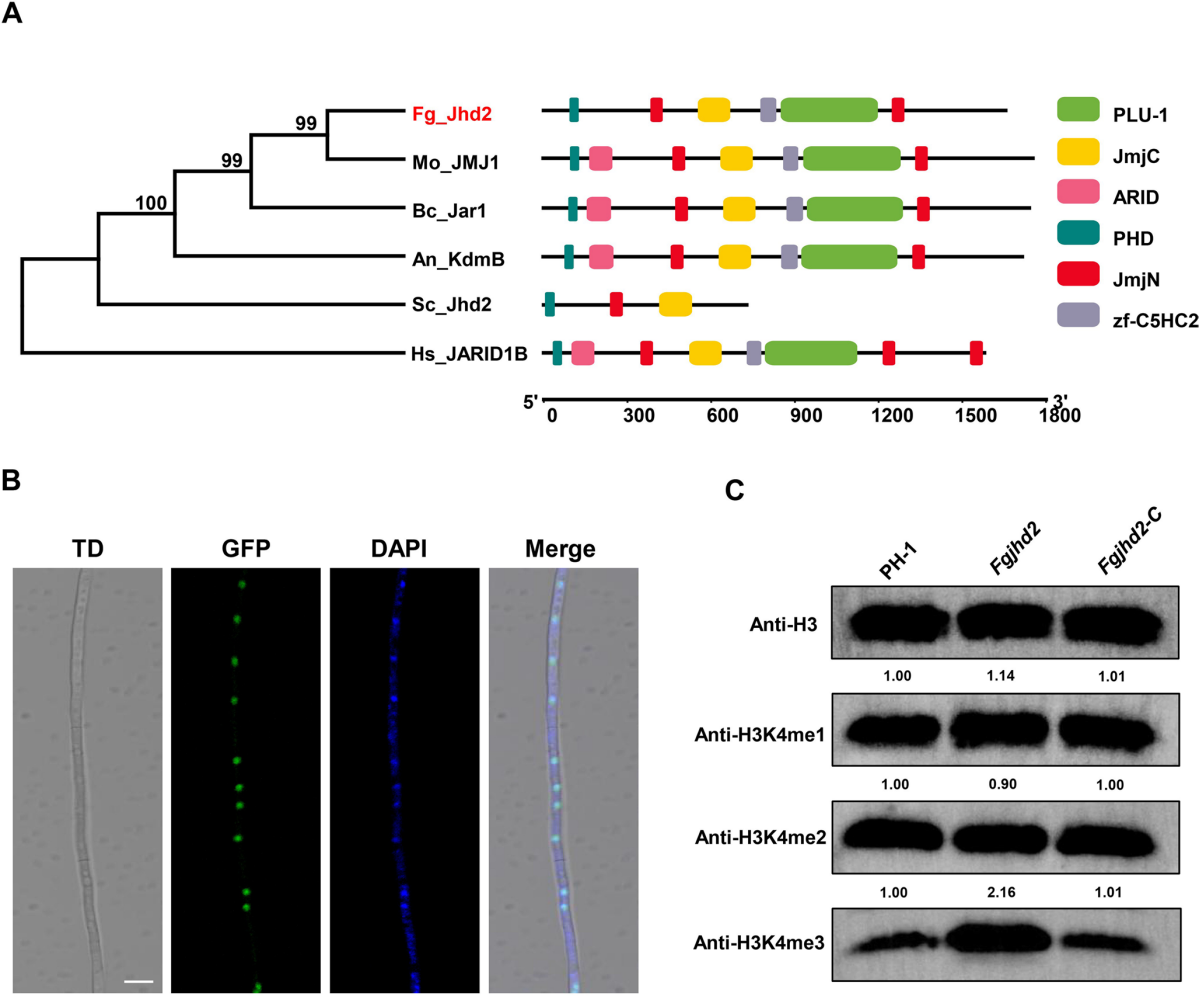
FgJhd2 is a conserved H3K4me3 demethylase. (A) Phylogenetic and conserved domain analysis of BcJar1 and its orthologs. The following protein sequences were used: *F. graminearum* Jhd2 (XP_011324456.1), *Magnaporthe oryzae* JMJ1 (XP_003712359.1), Botrytis cinerea Jar1 (XP_024546792.1), *Aspergillus nidulanus* KdmB (XP_681480.1), *Saccharomyces cerevisiae* Jhd2 (NP_012653.1) and *Homo sapiens* JARID1B (NP_001300971.1). (B) After staining with 4,6‐diamidino‐2 phenylindole (DAPI), the hyphae of the complementary strain *Fgjhd2*‐C are examined by laser confocal microscopy. Bars = 10 μm. (C) Western blot analysis of H3K4 demethylation activity of FgJhd2. Strains PH‐1, *Fgjhd2* and *Fgjhd2*‐C were cultured in liquid YEPD medium for 24 h. Total protein extracts were isolated from fresh hyphae and immunoblotted with antibodies detecting mono (me1), di (me2) and tri (me3) methylated H3K4 and total H3 (loading control). Bands were quantified using ImageJ software.

Bioinformatics analysis showed that the *FGSG_05855* gene of *F. graminearum* encodes a 1656‐amino‐acid protein that is an orthologue of *S. cerevisiae* Jhd2 (53% similarity), named FgJhd2. Similar to yeast Jhd2, FgJhd2 contains conserved JARID1‐group structural motifs, including an N‐terminal JmjN domain, a catalytic JmjC domain, two PHD finger domains, a C5HC2‐type zinc finger motif and a PLU‐1 domain (Figure 1A). FgJhd2 is within the JARID1 subfamily of histone demethylases (Figure 1A), which includes characterised orthologs from *Homo sapiens* , *Aspergillus nidulans*, *Botrytis cinerea* and *M. oryzae* . Notably, FgJhd2 exhibited higher sequence homology with MoJMJ1 from *M. oryzae* compared to other orthologs (Figure 1A).

To clarify the demethylase activity and biological function of FgJhd2, its gene replacement construct was constructed with the split‐marker approach using the hygromycin phosphotransferase (*hph*) cassette and transformed into the wild‐type strain PH‐1 (Cuomo et al. 2007) (Figure S1A). Deletion mutants *Fgjhd2* were verified by hygromycin resistance screening and PCR (Figure S1B). A complementary strain (*Fgjhd2*‐C) was generated by reintroducing the native *Fgjhd2* allele with its endogenous promoter into the *Fgjhd2* mutant. To enable subcellular localisation studies, a C‐terminal GFP fusion construct was incorporated into the complemented strain. Fluorescence microscopy combined with DAPI nuclear staining revealed distinct nuclear localisation of FgJhd2‐GFP in hyphae (Figure 1B).

Given the established role of JARID1‐family demethylases in H3K4 demethylation, we quantified histone methylation states using antibodies specific for mono‐ (H3K4me1), di‐ (H3K4me2) and tri‐methylated (H3K4me3) H3K4. Western blot analysis demonstrated a significant accumulation of H3K4me3, but not H3K4me1 or H3K4me2, in *Fgjhd2* vegetative hyphae relative to both wild‐type (PH‐1) and complemented (*Fgjhd2*‐C) strains (Figure 1C). This demethylase‐specific activity was conserved during sexual reproduction, mirroring the phenotype observed in vegetative growth. These findings align with previous reports of JARID1 orthologs such as AnKdmB ( *A. nidulans* ), MoJmj1 ( *M. oryzae* ) and BcJar1 ( *B. cinerea* ), collectively establishing FgJhd2 as a conserved JmjC‐domain‐dependent H3K4me3 demethylase in *F. graminearum*.

### 3.2 FgJhd2 Is Required for Vegetative Growth and Pathogenesis in *F. graminearum*

**FIGURE 2:**
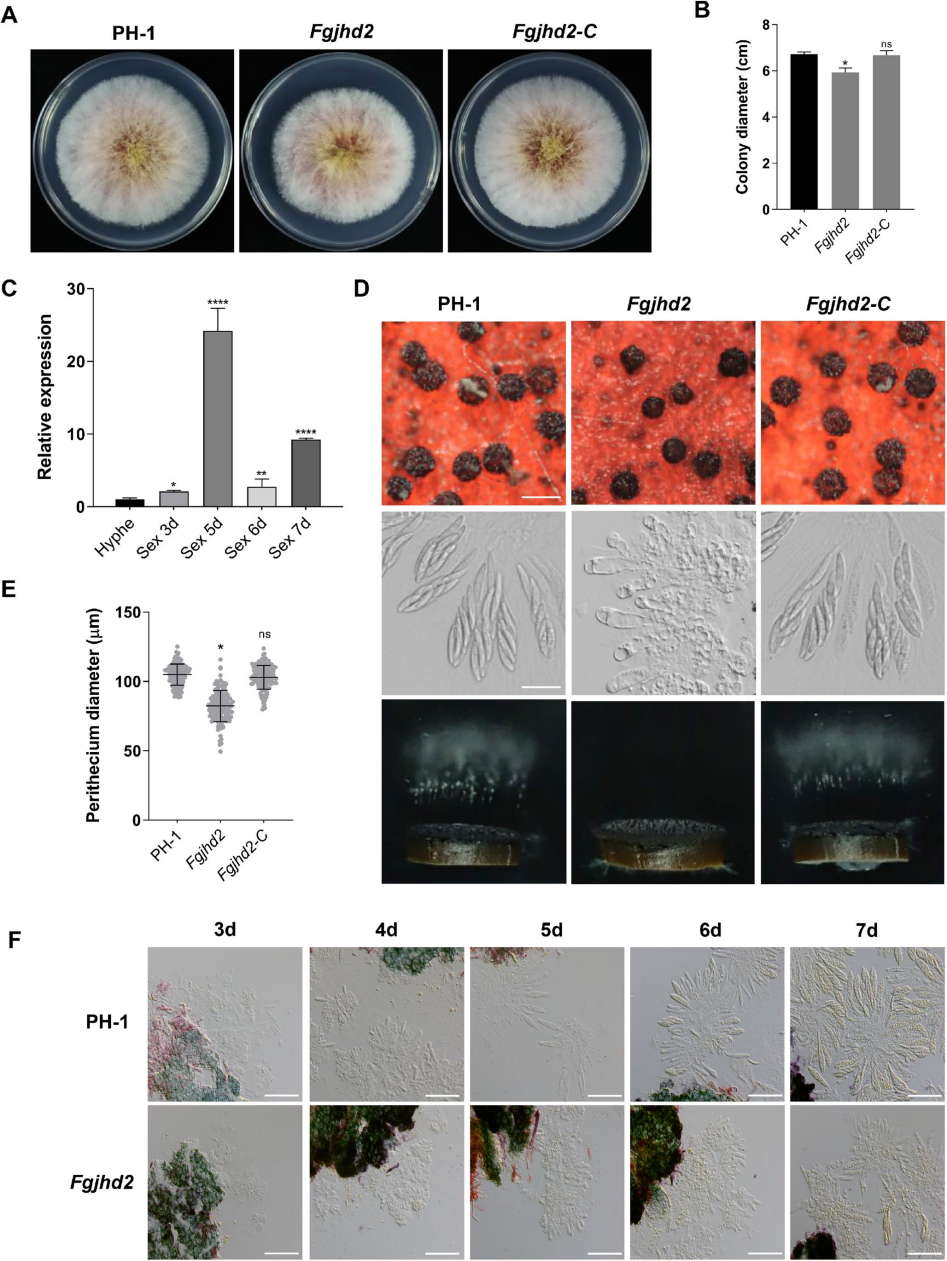
FgJhd2 is involved in vegetative growth, infection and sexual reproduction. (A) Colony morphology of the PH‐1, *Fgjhd2* and *Fgjhd2‐*C strains on PDA medium. Photographs were taken at 3 days after inoculation. (B) Comparison of colony diameters of the PH‐1, *Fgjhd2* and *Fgjhd2‐*C strains on PDA after 3 days. (C) The *Fgjhd2* mutant was defective in ascospore and asci. The expression level of *Fgjhd2* was examined using RNA isolated from vegetative hyphae cultured in YEPD medium for 12 h and from perithecia induced for 3, 5, 6 and 7 dpf. (D) Morphology of perithecia and cirri (white arrows), asci/ascospores and ascospore discharge of the PH‐1, *Fgjhd2* and *Fgjhd2‐*C strains on carrot agar. Photographs were taken at 7 days postfertilisation. Bar = 100 μm (Upper), bar = 20 μm (Bottom). (E) Comparison of perithecia diameters of the PH‐1, *Fgjhd2* and *Fgjhd2*‐C strains. Photographs were taken at 7 days postfertilisation. Measure the diameter of at least 100 perithecia per strain under a microscope. (F) Detection of asci and ascospores in perithecia of strains PH‐1 and the *Fgjhd2* mutant at 3, 4, 5, 6 and 7 dpf. Bar = 20 μm. Data was analysed with one‐way ANOVA, *p < 0.05; **p < 0.01; ****p < 0.0001, ns, not significant.

To determine whether FgJhd2 is indispensable for the vegetative growth of *F. graminearum*, PH‐1, the *Fgjhd2* mutant and complementary strain *Fgjhd2*‐C were cultured on PDA medium for 3 days. Compared with the PH‐1 strain, the growth of the *Fgjhd2* mutant was significantly reduced by 11.6% (Figure 2A,B) and there was no significant difference between the complemented strain *Fgjhd2*‐C and PH‐1. The *Fgjhd2* mutant was normal in conidiation and conidium morphology (Figure S2A,B), but it showed significantly reduced infectivity on wheat head, with a 44.1% decrease in disease index relative to the wild‐type strain (Figure S2C,D). These data indicated that FgJhd2 is required for vegetative growth and plant infection.

In addition, to investigate whether FgJhd2 regulates cell wall integrity and osmotic stress response in *F. graminearum*, we cultured strains PH‐1, *Fgjhd2* and *Fgjhd2*‐C for 3 days on PDA medium supplemented with cell wall damaging agents Congo red (CR) and sodium dodecyl sulfate (SDS), osmotic agent NaCl and oxidative stress agent H_2_O_2_. The *Fgjhd2* mutant showed enhanced sensitivity to 0.02% SDS but reduced sensitivity to 0.05% CR. There were no significant growth differences observed under 1.5 M NaCl or 20 mM H_2_O_2_ (Figure S3A,B). These findings indicate that FgJhd2 mediates cell wall integrity but not osmotic/oxidative stress tolerance.

### 3.3 FgJhd2 Is Essential for Sexual Development

We assayed *FgJHD2* expression in PH‐1 by qRT‐PCR with RNA isolated from nutrient mycelia cultured in YEPD for 12 h and from perithecia induced at 3, 5, 6 and 7 days post‐fertilisation (dpf). Compared to the hyphal stage, *FgJHD2* expression was upregulated during sexual development, peaking in perithecia at 5 dpf, suggesting its critical role in ascus and ascospore formation (Figure 2C). Deletion mutants of *Fgjhd2* formed smaller perithecia without cirrhi formation and spore firing at 7 dpf (Figure 2D,E). And when perithecia were examined, only a limited number of prematurely degraded asci were observed, with no mature ascospores detected (Figure 2D).

To elucidate the functional details of *Fgjhd2* during sexual development, we examined perithecia from 3 to 7 dpf (Figure 2F). The *Fgjhd2* mutant exhibited normal formation of ascogenous hyphae and croziers. However, at 5 dpf, elongated asci were rarely observed and ascogenous hyphae began degrading. By 7 dpf, although elongated asci could be detected, they contained no mature ascospores, accompanied by extensive degradation of ascogenous hyphae (Figure 2F). These results demonstrate that FgJhd2 is important for ascus formation and maturation, possibly by preventing premature degeneration of ascogenous hyphae and asci.

### 3.4 FgJhd2 Transcriptionally Regulates Key Genes Involved in Sexual Development

**FIGURE 3:**
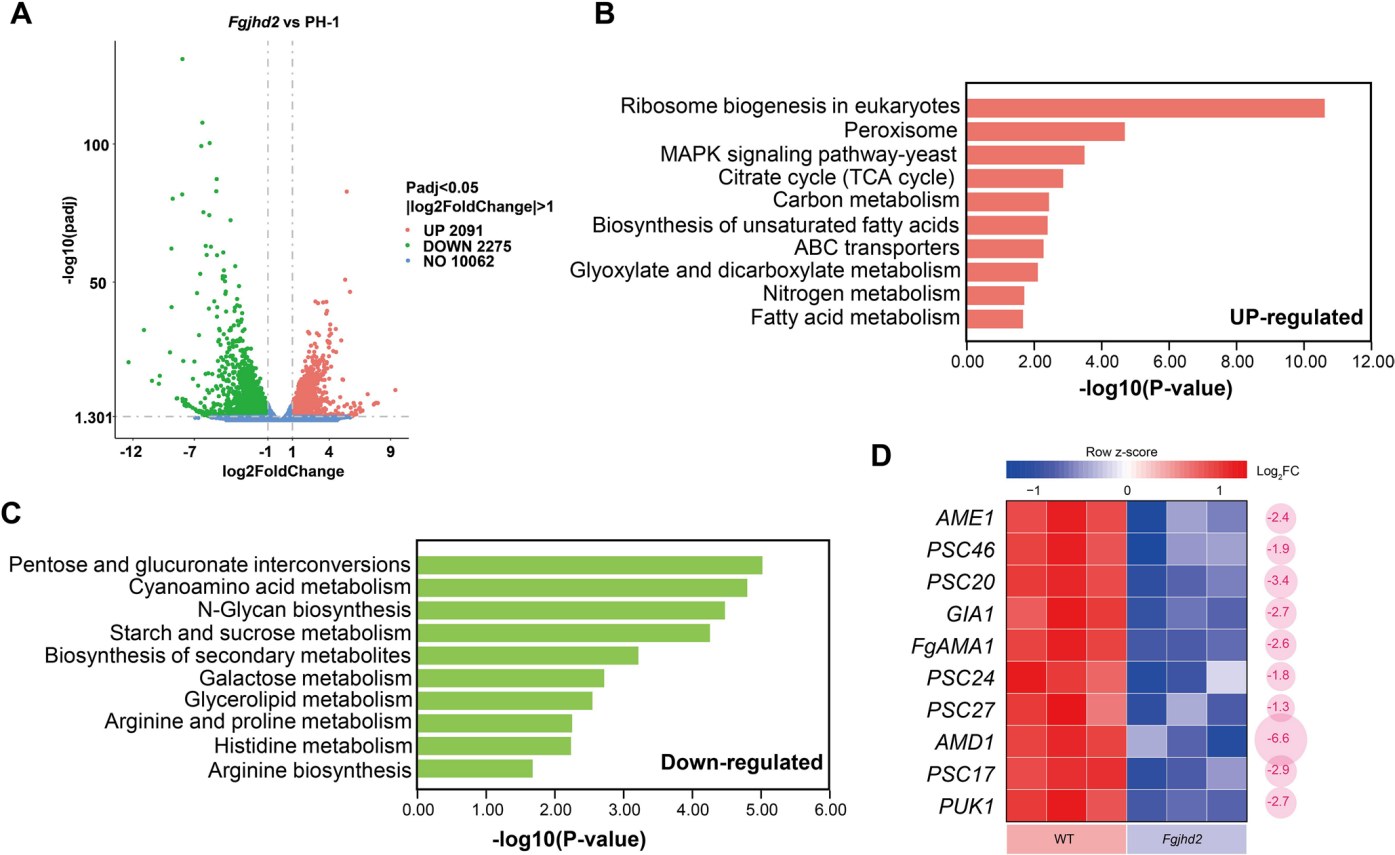
RNA‐seq analysis of PH‐1 and *Fgjhd2*. (A) Differentially expressed genes (DEGs) in *Fgjhd2* compared with the strain PH1. Up‐ and down‐regulated genes are highlighted with red and green points, respectively. Genes with a *p*‐value less than 0.05 and a fold change greater than 2 are considered significantly differentially expressed genes. (B) KEGG enrichment analysis of upregulated genes in *Fgjhd2* compared to PH‐1. (C) KEGG enrichment analysis of downregulated genes in *Fgjhd2* compared to PH‐1. (D) Heatmap analysis showed that eight genes known to be associated with perithecial development exhibited distinct changes in their expression patterns.

To identify genes that are transcriptionally regulated by FgJhd2 during sexual development in *F. graminearum*, we conducted RNA‐seq analysis with RNA isolated from perithecia collected from mating cultures at 3‐dpf. Compared to the wild‐type strain, the *Fgjhd2* mutant exhibited 4366 differentially expressed genes (DEGs), with 2091 genes up‐regulated and 2275 genes down‐regulated by at least two‐fold (Figure 3A). Among the up‐regulated genes, KEGG enrichment analysis revealed significant enrichment of pathways related to fatty acid metabolism, including peroxisome, the TCA cycle, biosynthesis of unsaturated fatty acids and glyoxylate and dicarboxylate metabolism (Figure 3B). This suggests that the deletion of *FgJHD2* may affect fatty acid synthesis and metabolism. Among the down‐regulated genes, KEGG analysis primarily showed enrichment in metabolic pathways such as pentose and glucuronate interconversions, cyanoamino acid metabolism, N‐glycan biosynthesis and starch and sucrose metabolism (Figure 3C). In ascomycetes, sexual development requires a lot of energy and fatty acids are a key resource to support this complex cellular process. Therefore, FgJhd2 may regulate sexual reproduction by participating in the regulation of carbon metabolism, particularly fatty acid metabolism.

Notably, among the DEGs down‐regulated in the *Fgjhd2* mutant, at least 10 of them are known to be important for the development of perithecium (e.g., *AME1*), ascogenous hyphae (e.g., *PSC46*), ascus (e.g., *AMD1*) and ascospore (e.g., *PUK1*) (Figure 3D). Their reduced expression might be responsible for the defects observed in the *Fgjhd2* mutant.

### 3.5 The Deletion of *FgJHD2* Maintains H3K4 Methylation

**FIGURE 4:**
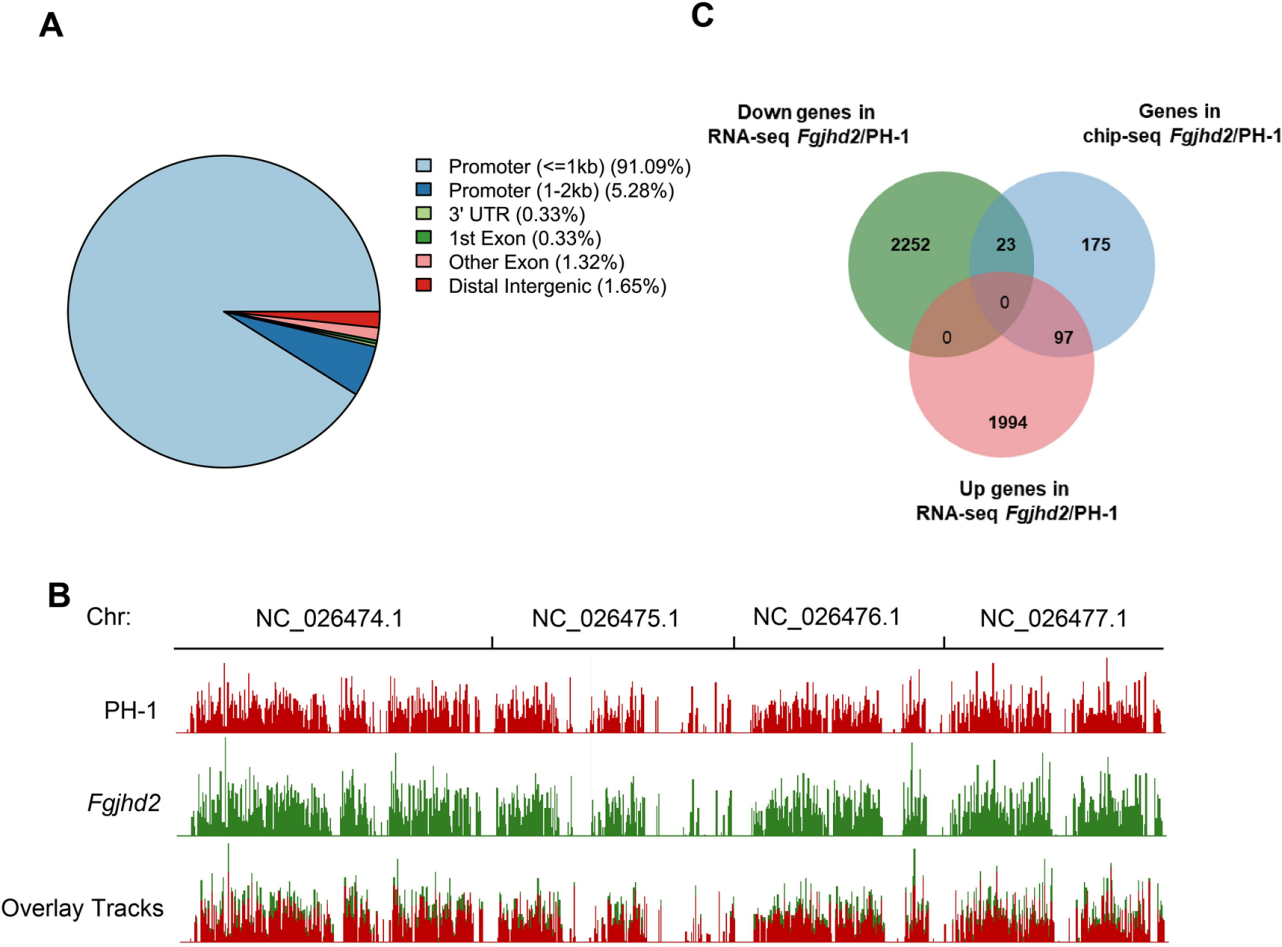
The overlapping genes between the unique ChIP peaks in *Fgjhd2* and the differentially expressed genes (DEGs) in RNA‐seq. (A) The pie chart shows the distribution of the ChIP peaks of *Fgjhd2* in different regions of the genome. (B) The genome browser image displays the distribution landscape of ChIP peaks for PH‐1 and *Fgjhd2* across the chromosomes. Overlay tracks were utilised in the Integrative Genomics Viewer. (C) The Venn diagram illustrates a statistically significant overlap between the unique ChIP peaks in *Fgjhd2* and the up‐regulated and down‐regulated DEGs.

Histone H3K4 methylation in eukaryotes is associated with transcriptional activation. We propose that *Fgjhd2* deletion mutant locks target genes in a transcriptionally active state, possibly through failed demethylation of H3K4me3. We subsequently compared the H3K4me3 methylation levels between the wild‐type and *Fgjhd2* strains using ChIP‐seq. In the sequencing results, we identified 3996 and 4115 peaks in the two replicates of the wild‐type, involving 3913 and 3890 genes, respectively. In the two replicates of *Fgjhd2*, we identified 3803 and 3838 peaks, involving 3752 and 3780 genes, respectively. Annotation of these enriched regions revealed that H3K4me3 is predominantly enriched at promoter regions, particularly near the transcription start site (Figure 4A,C and Figure S4). Comparative ChIP‐seq analysis revealed that H3K4me3 profiles in the *Fgjhd2* mutant exhibited similar genomic distribution patterns as wild‐type (WT) across all four chromosomes, but with significantly increased peak intensities (Figure 4B). This suggests that FgJhd2 regulates H3K4me3 abundance through demethylation.

By comparing the peak signals between the wild‐type and *Fgjhd2*, we identified 304 unique peaks in *Fgjhd2*, involving 295 genes. Integration with the DEGs identified in the RNA‐seq, we found 97 up‐regulated and 23 down‐regulated overlapping genes, respectively (Table S1). Among the 97 up‐regulated genes, KEGG analysis revealed significant enrichment in pathways related to propanoate metabolism, DNA repair and recombination proteins and phenylalanine, tyrosine and tryptophan biosynthesis (Table S1). Among the 23 down‐regulated genes, KEGG analysis revealed significant enrichment in pathways related to pentose and glucuronate interconversions, thiamine metabolism and arginine biosynthesis (Table S1).

### 3.6 FgMpf2 Acts Downstream of FgJhd2 to Regulate Sexual Development

**FIGURE 5:**
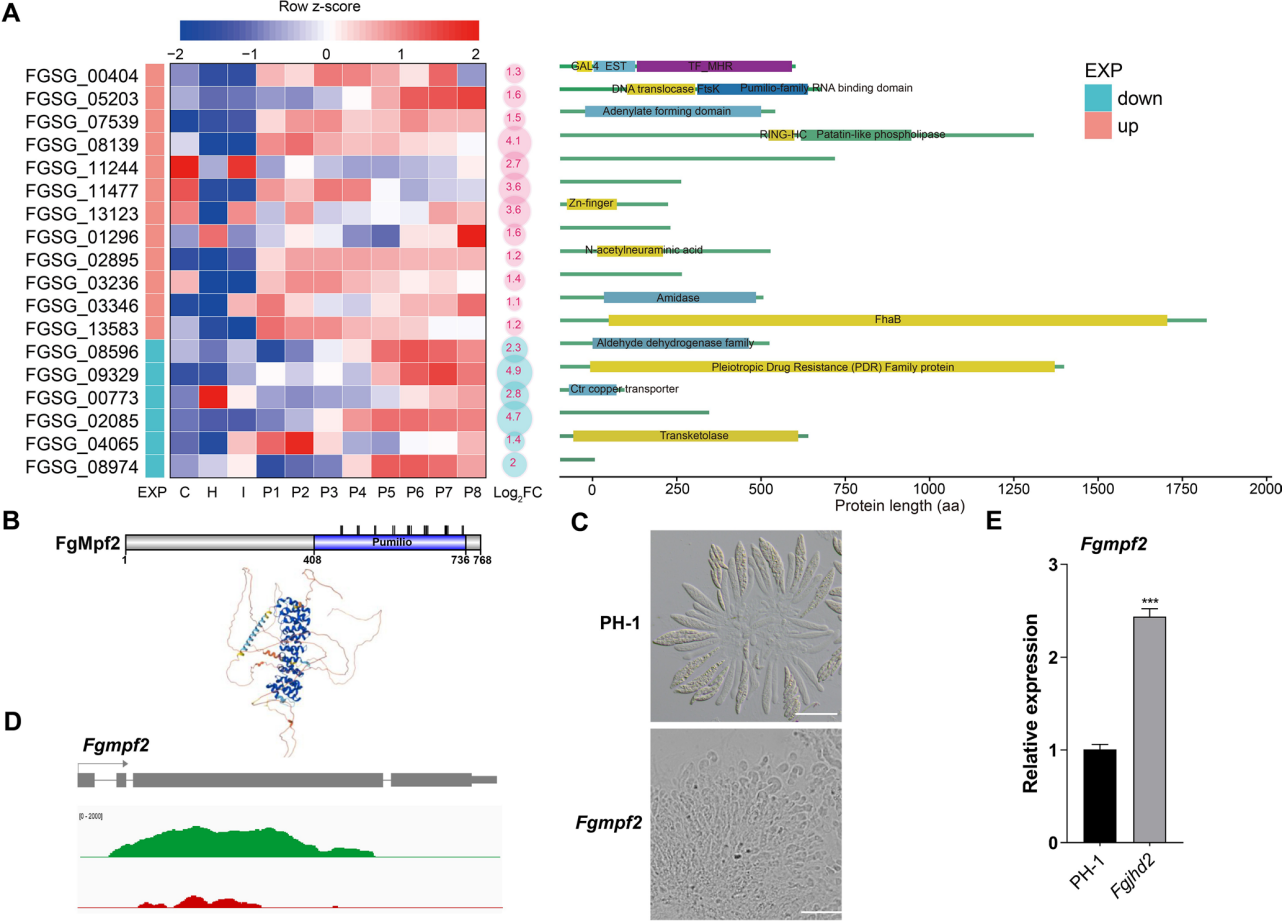
FgJhd2 regulates histone methylation and transcription of *Fgmpf2*, an RNA‐binding protein essential for sexual development in *F. graminearum*. (A) Heatmap analysis revealed distinct expression pattern changes in 18 related genes, together with schematic representations of their predicted protein domain architectures. (B) Predicted 3D protein structure of Fgmpf2 showing that the central region predominantly adopts α‐helical conformations, a common feature of Pumilio family proteins. (C) Phenotypic comparison of perithecia and asci between the wild‐type and *Fgmpf2* mutant. The *Fgmpf2* mutant formed immature perithecia that failed to produce mature asci and ascospores. Bar = 10 μm. (D) ChIP‐seq analysis showing histone methylation enrichment at the FGSG_05203 (Fgmpf2) locus in the *Fgjhd2* mutant compared with the wild‐type PH‐1. (E) qRT‐PCR validation revealed that the expression level of FGSG_05203 in *Fgjhd2* was significantly upregulated compared to the wild‐type PH‐1. Data was analysed with one‐way ANOVA, ***p < 0.001.

To identify FgJhd2‐targeted genes among the 120 candidate genes (97 up‐ and 23 down‐regulated), we performed comprehensive analyses including expression profiling, domain characterisation and differential expression evaluation (Table S1). Based on these criteria, we selected 18 high‐priority candidates, including FGSG_13123 (transcription factor) and FGSG_05203 (meiotic_coiled‐coil_protein_2) for functional validation via gene knockout (Figure 5A). The deletion mutants were examined for defects in vegetative growth, perithecia formation and asci/ascospores maturation. The deletion mutants of all tested genes except FGSG_08139 showed normal vegetative growth, while the *FGSG_08139* mutant exhibited a slower growth rate (Figure S6A,B). The deletion of another 3 genes resulted in defects in different stages of sexual development. The *FGSG_13123* deletion mutant produced significantly fewer perithecia, but ascospores in the surviving perithecia showed no apparent morphological defects (Figure S6A). Deletion mutant of *FGSG_05203* developed normalised perithecia without typical asci (Figure S6A). When examined 7 dpf perithecia, scattered ascospores but fewer intact asci were observed in those of the *FGSG_02085* mutant (Figure S6A).

Sequence alignment revealed that FGSG_05203 is homologous to the *Schizosaccharomyces pombe* meiotic Pumilio family RNA‐binding protein SpMpf2 and was therefore designated FgMpf2 (Figure S5 and Figure 5B). Consistent with characteristic RNA‐binding architectures observed in Pumilio family proteins. The *FgMPF2* encodes a 768 amino acid protein that contains a well‐conserved Pumilio domain and 28 RNA binding sites and structural modelling reveals predominant α‐helical organisation in its central region (Figure 5B), The *Fgmpf2* mutant formed excessively elongated ascogenous hyphae and croziers, but its croziers did not produce asci at 7 dpf (Figure 5C). ChIP‐seq analysis revealed elevated H3K4me3 levels at the FGSG_05203 locus in *Fgjhd2* mutants compared to wild‐type (Figure 5D), which may implicate FgJhd2 in modulating H3K4me3 levels at this genomic region. The H3K4me3‐enriched chromatin state in *Fgjhd2* functionally translated to increased transcriptional output, with both RNA‐seq and qPCR validation showing concordant *FgMPF2* up‐regulation (Figure 5E). Collectively, these results support a model in which *FgMPF2* is negatively regulated by FgJhd2 and may participate in the developmental processes influenced by FgJhd2 in *F. graminearum*.

### 3.7 *FgMPF2* Overexpression Suppresses Transcript Levels of Three Key Genes Involved in Ascosporogenesis

**FIGURE 6:**
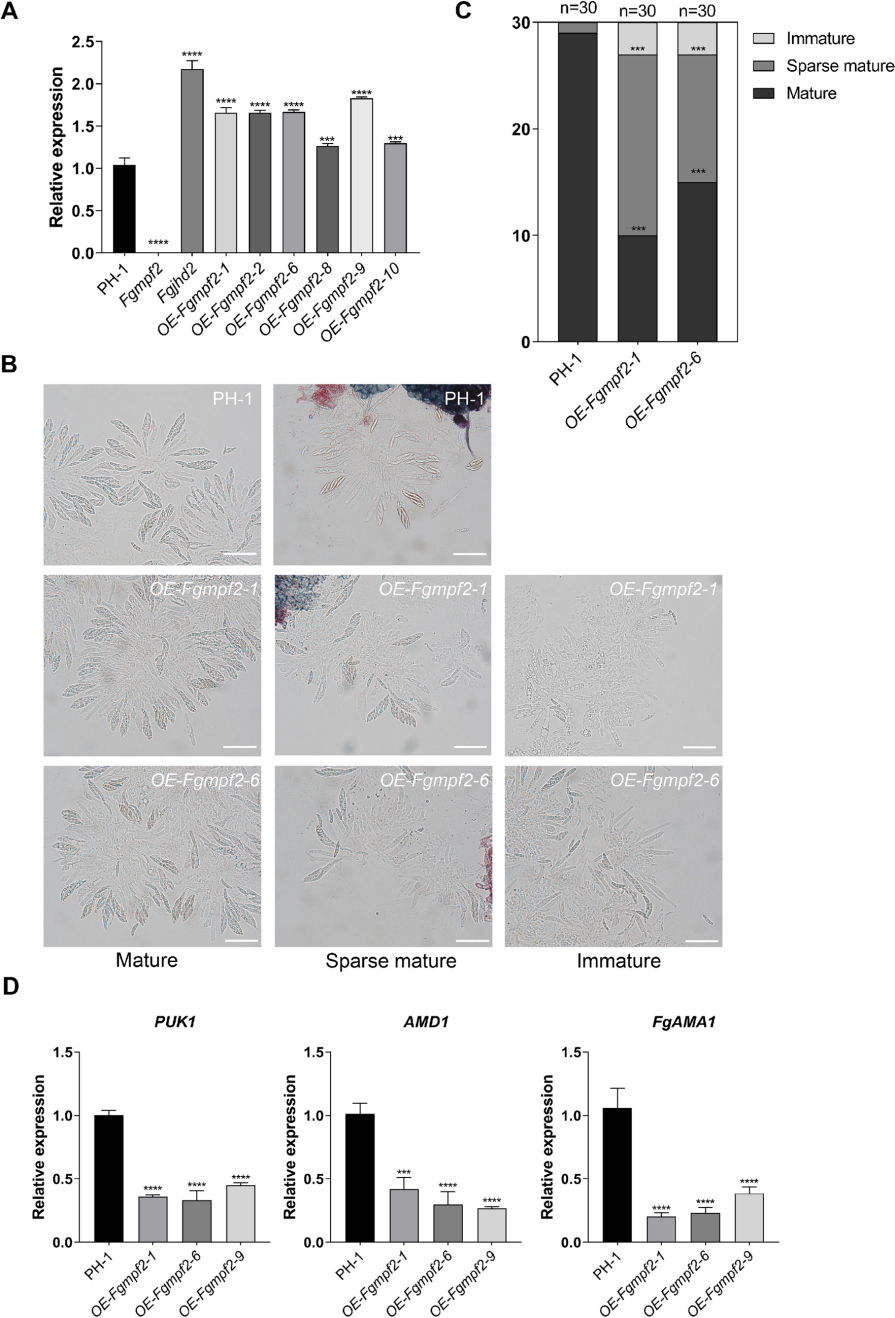
Overexpression of *Fgmpf2* affects perithecial maturation and ascospore development in *F. graminearum*. (A) qRT‐PCR analysis of *Fgmpf2* expression in the wild‐type strain (PH‐1), the *Fgjhd2* mutant and *Fgmpf2* overexpression transformants (*OE‐Fgmpf2*). (B) Microscopic observation of asci and ascospores in PH‐1 and OE‐Fgmpf2 transformants 7 days postfertilisation. *OE‐Fgmpf2* transformant ascospores exhibit developmental variations, showing normal, reduced and abnormal ascospores. Bar = 20 μm. (C) Statistical analysis of morphology in ascomata and ascospores of PH‐1 and *Fgmpf2* overexpressing strains. (D) Expression analysis of *PUK*, *AMD1* and *FgAMA1* in the *OE‐Fgmpf2* strain. Data was analysed with one‐way ANOVA, ***p < 0.001; ****p < 0.0001.

To further examine the regulatory relationship between FgJhd2 and FgMpf2, *Fgmpf2* overexpression strains were constructed under the control of the constitutive promoter (Figure 6A). qRT‐PCR analysis showed that transcript levels of *FgMPF2* in these strains (*OE‐Fgmpf2‐1*, *OE‐Fgmpf2‐6*) increased by less than twofold compared with the wild‐type PH‐1, indicating a relatively modest upregulation and suggesting that *FgMPF2* expression is under tight regulatory control. In wild‐type strains, the majority of fascicles of asci within 5‐dpf perithecia exhibited elongated asci, while only 15% contained non‐elongated asci, whereas *OE‐Fgmpf2* strains showed a significant retention of non‐elongated asci (35% of ascus fascicles) (Figure S7A,B). Compared to wild‐type strains where nearly all ascus fascicles showed uniform maturation (97% with fully mature ascospores), the *OE‐Fgmpf2* strains exhibited three distinct states: (1) fascicles containing predominantly mature ascospores, (2) fascicles with only sparse mature asci and (3) fascicles displaying minimal to no mature ascospores. Notably, the latter two defective states accounted for the majority of observed cases in mutant strains, whereas wild‐type perithecia never displayed the complete maturation failure seen in type 3 (Figure 6B,C). Relatively few fascicles of asci with mature ascospores were observed in 7‐dpf perithecia of *OE‐Fgmpf2* strains, indicating impaired ascus and ascospores maturation. Collectively, our data suggest that FgJhd2 influences ascus/ascospore maturation, at least in part, through its impact on *FgMPF2* expression.

A plausible model is that FgJhd2 loss activates *FgMPF2* expression, with excess FgMpf2 protein potentially triggering RNA degradation of sexual development genes—thereby disrupting normal maturation. To test this hypothesis, eight DEGs (*AME1*, *PSC46*, *PSC20*, *GIA1*, *FgAMA1*, *AMD1*, *PUK1* and *FGSG_02085*) down‐regulated in the *Fgjhd2* mutant were selected for qRT‐PCR assays in mating cultures of *OE‐Fgmpf2*. Compared to the wild‐type strain, three of them, *FgAMA1*, *AMD1* and *PUK1*, had significant downregulation in *OE‐Fgmpf2* strains (Figure 6D), *AME1*, *PSC46*, *PSC20*, *GIA1* and FGSG_02085 showed no substantial effects (Figure S8). Taken together, these findings suggest that elevated FgMpf2 levels in *Fgjhd2* mutants correlate with reduced transcript abundance of key sexual development‐related genes, which may contribute to the observed defects during *F. graminearum* sexual reproduction. Given FgMpf2's RNA‐binding and well‐conserved Pumilio domain, altered RNA stability could be involved, though direct mechanistic evidence remains to be established.

## 4 Discussion

In ascomycete fungi, histone methylation plays a significant role in processes such as hyphal growth, development and pathogenic infection (Zhang and Tao 2025). *F. graminearum* prioritises reproduction over survival under resource trade‐off scenarios, exhibiting a reproduction‐biased pathogenic strategy (Qi et al. 2024). Consequently, disease management should target reproductive regulation to disrupt its life cycle. Histone methylation is a dynamic process regulated by histone methyltransferases and histone demethylases and histone methyltransferases have been confirmed to be involved in regulating sexual reproduction in *F. graminearum*. The deletion of the histone methyltransferase KMT6 in *F. graminearum* completely abolishes the sexual reproduction process (Adpressa et al. 2019). However, the function of histone demethylases in sexual reproduction has yet to be explored. In this study, we found that the absence of FgJhd2 caused loss of H3K4me3 and severe defects in vegetative growth, perithecia, ascogenous hyphae and asci development. Our RNA‐seq analysis identified 4366 DEGs in the *Fgjhd2* mutant, underscoring its broad regulatory influence. Among the DEGs down‐regulated in the *Fgjhd2* mutant, we observed several key regulators of sexual development, including: *AME1*, *PSC46*, *FgAMA1*, *AMD1* and *PUK1* etc. *AME1* plays a crucial role in perithecium morphogenesis (Feng et al. 2024), with the *ame1* mutant exhibiting reduced perithecium size similar to that observed in *Fgjhd2* strains. *PSC46*, *PSC20*, *GIA1*, *PSC24*, *AMD1* and *PSC27* are all important for ascogenous hyphae development (Ding et al. 2023; Qi et al. 2024). Particularly, both *psc46* and *Fgjhd2* mutants exhibit identical premature degradation phenotypes in ascogenous hyphae and asci development, indicating *FgJHD2* may act upstream of *PSC46* in this degradative pathway. *FgAMA1*, *PSC17* and *PUK1* are found to be essential for ascosporogenesis and/or ascus maturation (Liu et al. 2016; Qi et al. 2024; Cao et al. 2017; Hao et al. 2019). Therefore, the downregulation of these genes could be responsible for the defects observed in the *Fgjhd2* mutant.

**FIGURE 7:**
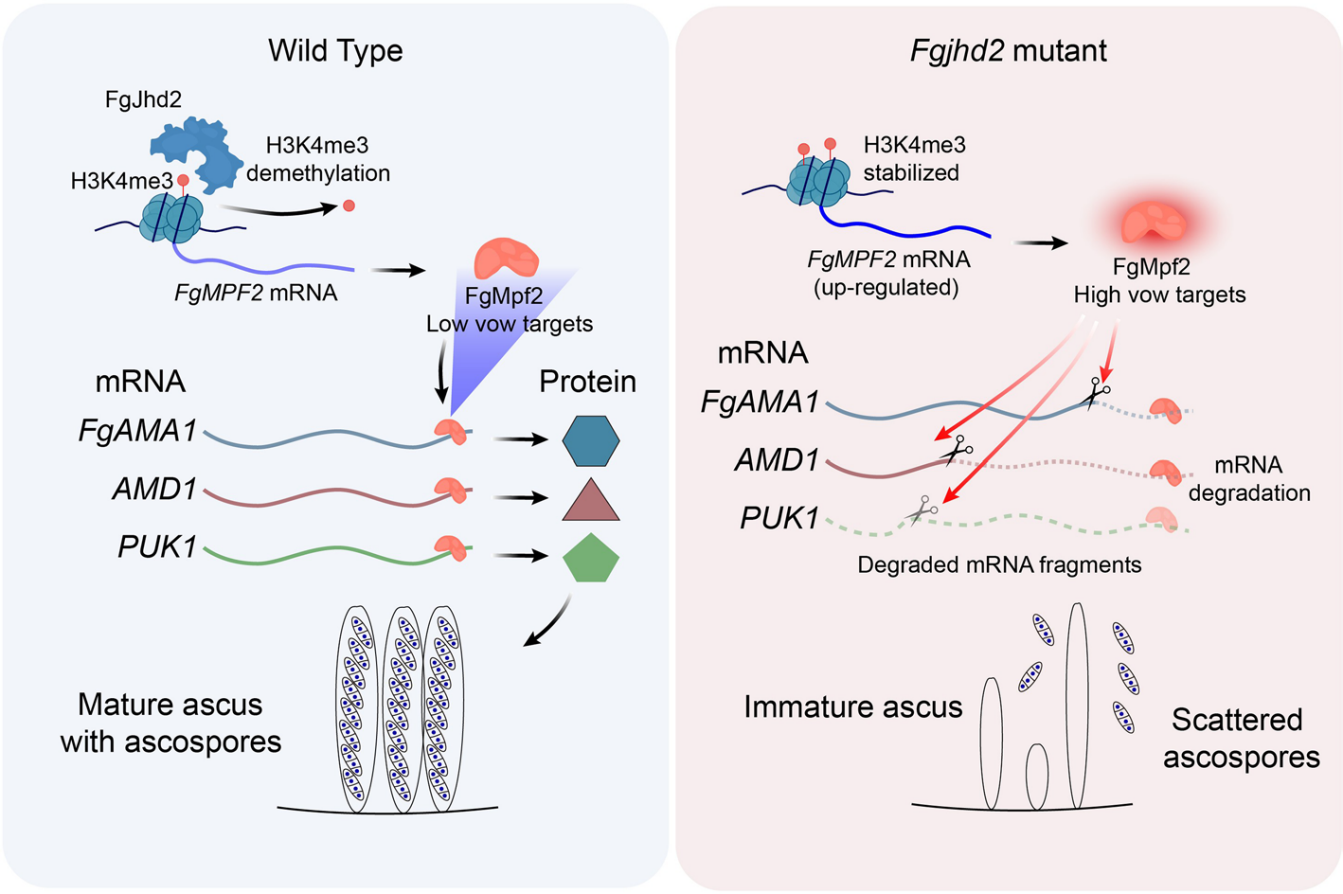
Model illustrating the regulatory role of FgJhd2 in controlling Fgmpf2 during sexual development in *F. graminearum*.

Since histone H3K4 methylation typically associates with transcriptional activation (Park et al. 2026), the loss of FgJhd2 (a demethylase) was expected to sustain methylation and up‐regulate target genes. Thus, the observed down‐regulation suggests an indirect mechanism, implicating an FgJhd2‐regulated transcriptional repressor upstream of these developmental genes. Strikingly, integrated ChIP‐seq and RNA‐seq analyses identified *FgMPF2* as a negatively regulated target of FgJhd2, with increased H3K4me3 enrichment at its locus correlating with elevated transcript levels. *FgMPF2* encodes a conserved Pumilio domain protein, homologous to yeast Puf3, which accelerates mRNA degradation of its targets (Olivas and Parker 2000; Jackson et al. 2004; Garrido‐Godino et al. 2021). The Pumilio family proteins are ubiquitously present across eukaryotes and generally bind to the 3′ untranslated regions of single stranded RNA targets in a sequence specific manner and destabilise them (Nishanth and Simon 2020). Based on its sequence similarity and domain architecture, FgMpf2 is best described as a putative Pumilio‐family RNA‐binding protein. We hypothesised that *FgMPF2* overexpression could destabilise mRNAs of sexual development genes, recapitulating the *Fgjhd2* mutant's defects. Indeed, *OE‐Fgmpf2* strains exhibited severely impaired ascus maturation, partially mimicking the phenotype of *Fgjhd2*. Strikingly, *OE‐Fgmpf2* strains recapitulated the transcriptional signature of *Fgjhd2*, with *FgAMA1*, *AMD1*, *PUK1* significantly down‐regulated. These findings support a model where dynamic H3K4me3 modulation integrates with RNA stability control to ensure precise gene expression during sexual reproduction (Figure 7). The conserved domains and homologous proteins identified in this study suggest that the FgJhd2‐FgMpf2‐associated regulatory pathway may be evolutionarily conserved in fungal sexual development, although the conservation of the complete regulatory mechanism remains to be further investigated.

The *Fgjhd2* deletion mutant exhibited not only defective ascus and ascospore development but also smaller perithecia, reduced ascogenous hyphae and premature degeneration of ascogenous hyphae and asci, which phenotypes are absent in *OE‐Fgmpf2* strains. We propose two non‐mutually exclusive mechanisms: (1) The relatively modest *FgMPF2* overexpression (~1.6‐fold) in our OE strains may be below the threshold required to phenocopy *Fgjhd2*'s elevated levels (~2.2‐fold) (Figure 6A), implying dose‐dependent control of target mRNA selection during RNA degradation. (2) Discrete developmental regulation may occur through alternative pathways modulating key genes (*AME1*, *PSC46*, *GIA1*, *PSC27*, etc.), necessitating deep integrated analysis of available RNA‐seq and ChIP‐seq datasets. H3K4 methyltransferase Set1 and Jhd2 co‐regulate transcription at some target genes, such as *SER3* down‐regulated in either *set1* or *jhd2* mutant (Ramakrishnan et al. 2016). Set1 and Jhd2, via modulating H3K4 methylation‐demethylation, together control chromatin dynamics during various facets of transcriptional regulation (Ramakrishnan et al. 2016). Unlike the canonical target *FgMPF2*, whose expression is suppressed by FgJhd2‐mediated demethylation, *AME1* and other developmentally critical genes may be under direct control of FgJhd2‐mediated chromatin remodelling.

Functional analyses indicate that FgMpf2 is required for ascus differentiation and *OE‐Fgmpf2* impaired ascus maturation, suggesting that FgMpf2 likely acts as a downstream effector of FgJhd2 during late stages of sexual development. In addition, given the extensive transcriptional reprogramming observed in the *Fgjhd2* mutant, it is likely that FgJhd2 coordinates sexual development through multiple downstream targets, of which FgMpf2 represents one key component within a broader regulatory network. Gene expression is coordinately regulated through multi‐layered mechanisms, including epigenetic modifications (on genomic DNA and histones) and RNA degradation. In recent years, it has become increasingly recognised that RNA decay is of no less importance than RNA synthesis and contributes to regulating the RNA level to control gene expression (Tatosyan et al. 2020). We found that FgMpf2, a Pumilio‐family RNA decay factor, is essential for ascus formation and meiosis in *F. graminearum*. Furthermore, the expression of *FgMPF2* is tightly regulated, as both deletion and overexpression lead to severe developmental defects. In *F. oxysporum*, *FonPUF1* (Pumilio 1) regulates virulence on watermelon by binding to the 3′ UTR of a set of putative virulence‐related genes to coordinate the expression (Gao et al. 2023). However, whether the reduced transcript levels arise from altered transcription, changes in RNA stability, or direct binding of FgMpf2 to these transcripts remains to be determined. Future studies, including RNA‐binding and transcript stability assays, will be required to define the underlying molecular mechanism.

In summary, our study establishes FgJhd2 as a central regulator of sexual development in *F. graminearum*, governing perithecium development, ascus and ascospores maturation through H3K4me3 demethylation. Mechanistically, FgJhd2 fine‐tunes the expression of the RNA‐binding protein FgMpf2 by modulating H3K4me3 levels, thereby controlling the mRNA abundance of key developmental genes (*FgAMA1*, *AMD1*, *PUK1*). These findings unveil a previously unrecognised epigenetic‐RNA stability checkpoint that ensures faithful sexual reproduction. Future studies should (1) test FgMpf2 directly binds to targets' mRNA via RIP or EMSA, (2) explore whether FgJhd2 regulates additional post‐transcriptional effectors and (3) investigate whether this regulatory axis influences fungal pathogenicity. Such efforts would deepen our understanding of how chromatin dynamics and RNA metabolism converge to control fungal development.

## 5 Materials and Methods

### 5.1 Strains and Growth Conditions of *F. graminearum*

The *F. graminearum* wild‐type strain PH‐1 (NRRL 31084) and all mutant/transformants generated in this study were grown on potato dextrose agar (PDA) plates at 25°C in darkness. The colony diameter and morphology were measured after 3 days of growth on PDA plates and conidia were measured after 5 days of growth in CMC medium. The mycelia grown for 24 h in yeast extract peptone glucose (YEPD) medium (3 g yeast extract, 10 g peptone, 20 g D‐glucose) were used for fluorescence observation. To determine the mutant defect in the stress response, final concentrations of 0.02% sodium lauryl sulfate (SDS), 0.05% Congo red (CR), 1.5 M NaCl and 20 mM H_2_O_2_ were added to the PDA plates. For sexual development, mycelia were grown on carrot agar medium for 5 days, then overwhelmed aerial hyphae with sterile 0.1% Tween 20 and incubated under black light at 25°C. Perithecium formation, cirrus production, ascus development and ascospore discharge were examined after incubation for 1–2 weeks.

### 5.2 Plant Infection

For infection assays with wheat spikes of cultivar Fielder, conidia were harvested in CMC medium cultured for 5 days and resuspended to 1 × 10^6^ conidia/ml with sterile ddH_2_O. For each spike, 10 μL of conidia suspension was inoculated from the 5th spikelet at the base of the spike. Fourteen days after infection, the onset of symptoms was examined and the disease index was counted. The experiment was independently repeated three times, with similar results each time.

### 5.3 RNA Extraction and Quantitative Real‐Time PCR (qRT‐PCR) Analysis

Total RNA was extracted from the vegetative mycelia or perithecia of wild‐type and mutant strains using the SPARKeasy Plant RNA Kit (SparkJade, Shandong, China). Subsequently, reverse transcription was performed to obtain cDNA using the SPARKscript II All‐in‐one RT SuperMix for qPCR (SparkJade, Shandong, China). The *β‐tubulin* gene was used as an endogenous reference gene and the expression levels of the target genes were quantified by qRT‐PCR using the ChamQ Universal SYBR qPCR Master Mix (Vazyme, Shandong, China). Finally, the relative gene expression levels were calculated using the 2^−ΔΔCT^ method.

### 5.4 The Construction of Gene Knockout, Complementation and Overexpression Strains

In order to clarify the function of *Fgjhd2*, this gene was deleted in *F. graminearum* PH‐1. The split‐marker system was used to replace the *Fgjhd2* gene with the hygromycin resistance gene (*hyg*). A PEG‐mediated protoplast transformation method was used to transfer DNA fragments into the *F. graminearum*. Knockout mutants need to be verified by hygromycin selection and PCR assays.

For complementary assays, the self‐promoter and ORF (without stop codon) of *Fgjhd2* were amplified and ligated to the pKNT vector by seamless cloning. GFP fluorescent protein was fused at the C‐terminus of FgJhd2. The pKNT‐FgJhd2‐GFP vector was transformed into the *Fgjhd2* knockout mutant to generate complementary strains.

The overexpression strain was constructed using the pDL2‐GFP vector. The full‐length gene (without the stop codon) was amplified and ligated into the vector through seamless cloning. Subsequently, the recombinant vector was transformed into the wild‐type strain PH‐1 using the PEG‐mediated protoplast transformation system. Finally, the transcript level in the overexpression strain was detected by qRT‐PCR.

### 5.5 Western Blotting Analysis

For western blot analysis, 0.2 g of perithecium cultured under black light for 3 days was collected. Proteins were acid extracted as previously described (Feng et al. 2024). Frozen samples of perithecium were ground and added to RIPA Lysis Buffer (P0013C, Beyotime, Shanghai). The homogenates were centrifuged (13,500 rpm, 10 min, 4°C) and the supernatant was collected. Fifteen micrograms of proteins were used for SDS‐Page and western blotting. H3 methylation was probed with polyclonal anti‐H3K4me1 (ab8895, Abcam, Cambridge, UK), anti‐H3K4me2 (ab7766) and anti‐H3K4me3 (ab8580). Detection with monoclonal anti‐H3 (HL102, TransGen Biotech, Beijing, China) was used as a control.

### 5.6 ChIP‐Seq Analysis

The chromatin immunoprecipitation (ChIP) experiment was performed according to the protocol provided by Gendrel et al. (2005) with appropriate modifications. Perithecia materials at 3 dpf from the PH‐1 and *Fgjhd2* mutant strains were collected, with two biological replicates each for the WT and mutant strains. The fragmented chromatin was incubated with Anti‐H3K4me3 (ab8580, Abcam, Cambridge, UK) for downstream experiments. The ChIP‐seq analysis was conducted by Wuhan IGENEBOOK Biotechnology Co. Ltd.

### 5.7 RNA‐Seq Analysis

Perithecia of PH‐1 and *Fgjhd2* mutants cultured on carrot agar medium for 3 days were used to extract total RNA, with three biological replicates provided for each strain. The integrity of the extracted total RNA was quality‐controlled using the Agilent 2100 bioanalyzer system (Agilent Technologies, CA, USA). Subsequently, mRNA was purified from the total RNA using poly‐T oligonucleotide‐attached magnetic beads. The cDNA library was then generated through cDNA synthesis, purification and PCR amplification. Finally, the resulting libraries were sequenced using the Illumina Novaseq platform at Novogene Bioinformatics Technology (Beijing, China). The sequencing reads were aligned to the reference genome of *F. graminearum* PH‐1 using Hisat2 v2.0.5. Differential expression analysis was performed using the DESeq2 R package (version 1.20.0), with genes having a *p*‐value ≤ 0.05 and |log_2_(FoldChange)| ≥ 1 considered as differentially expressed.

## Author Contributions

**Zhaomei Qi:** writing – original draft, writing – review and editing, resources, project administration, funding acquisition, validation, visualization. **Yuan Chen:** project administration, resources, writing – review and editing, writing – original draft, visualization, validation, funding acquisition. **Yingqi Chen:** investigation, validation, visualization. **Shisheng Chen:** writing – review and editing. **Liqiang Yao:** investigation, methodology, writing – review and editing, writing – original draft, validation, visualization. **Xiwen Liao:** conceptualization, methodology, software, data curation, investigation, writing – review and editing, validation, visualization. **Jie Wang:** conceptualization, methodology, validation, visualization.

## Conflicts of Interest

The authors declare no conflicts of interest.

## Supporting information


**Figure S1:** Targeted deletion of *FgJHD2* in *F. graminearum*. (A) Through homologous recombination, the coding sequence of *Fgjhd2* was replaced with the hygromycin resistance gene (*hyg*). Using double‐ligation PCR, the upstream and downstream homologous arms (HY and YG) of *Fgjhd2* were ligated with the *hyg* fragment to construct a knockout fragment for fungal transformation. (B) PCR identification results for the knockout mutant strains. Validation was performed using specific primers located on the outer side of the homologous arms to detect gene replacement. The successfully obtained knockout mutant strains (*Fgjhd2‐1* and *Fgjhd2‐2*) exhibited expected amplification bands, while no *hyg* fragment signal was detected in the wild‐type strain (PH‐1).
**Figure S2:** FgJhd2 no affects the conidial morphology or production and of *F. graminearum*. (A) Conidium length of WT, *Fgjhd2* mutant strain and complementary strain. (B) Conidium production of WT, *Fgjhd2* mutant strain and complementary strain. Bars represent the standard deviation. (C) Virulence assays on flowing wheat heads with conidia of the PH‐1, *Fgjhd2* and *Fgjhd2‐*C strains. Photographs were taken at 14 days after inoculation (dpi). (D) Disease index counted after 14 dpi. Data was analysed with one‐way ANOVA, *p < 0.05, ns, not significant.
**Figure S3:** FgJhd2 affect the growth and stress response of *F. graminearum*. (A) Wild‐type (PH‐1) and *Fgjhd2* mutant strains were cultured on potato dextrose agar (PDA) plates supplemented with 0.02% SDS, 0.05% Congo red, 1.5 M NaCl, 20 mM H_2_O_2_ at 25°C for 3 days. (B) Colony diameter of the strains on PDA medium. Data was analysed with two‐way ANOVA, *p < 0.05, ns, not significant.
**Figure S4:** Genome‐wide distribution of ChIP‐seq signals in PH‐1 and the *Fgjhd2* mutant.
**Figure S5:** Sequence alignment analysis revealing that Fgmpf2 shares high similarity with the meiotic Pumilio family RNA‐binding protein SpMpf2 from *S. pombe*.
**Figure S6:** Analysis of different mutant phenotypes. (A) Colony morphology of 18 gene deletion mutants selected from transcriptomic clustering after 3 days of incubation on PDA and perithecial, ascus and ascospore development observed on carrot agar at 7 dpf. Bar = 200 μm (Upper), bar = 20 μm (Bottom). (B) Comparison of colony diameters of the PH‐1 and 18 gene deletion mutants strains on PDA after 3 days. Data was analysed with one‐way ANOVA, *p < 0.05, ns, not significant.
**Figure S7:** Overexpression of *Fgmpf2* affects perithecial maturation and ascospore development in *F. graminearum*. (A) Microscopic observation of asci and ascospores in PH‐1 and *OE‐Fgmpf2* transformants 5 days postfertilisation. Bar = 20 μm. (B) Statistical analysis of morphology in ascomata and ascospores of PH‐1 and *Fgmpf2* overexpressing strains. Data was analysed with one‐way ANOVA, *p < 0.05, ***p < 0.001.
**Figure S8:** Expression analysis of *FGSG_02085*, *GIA1*, *PSC46, AME1* and *PSC20* in the *OE‐Fgmpf2* strain. Data was analysed with one‐way ANOVA, *p < 0.05, ns, not significant.

**Table S1:** Expression profiling and differential expression analysis of 120 candidate genes.

## Data Availability

Transcriptome data of *F. graminearum* have been deposited in the NCBI sequencing read archive BioProject (PRJNA1405148) (https://www.ncbi.nlm.nih.gov/bioproject/PRJNA1405148/). ChIP‐Seq data of *F. graminearum* read archive BioProject (PRJNA1405792) (https://www.ncbi.nlm.nih.gov/bioproject/PRJNA1405792/).
