# Supplementary material for: FgJhd2 Modulates FgMpf2 Expression via H3K4 Demethylation and Influences Sexual Development in Fusarium graminearum
Source: Environ Microbiol. 2026 Jun 18;28(6):e70354. doi: 10.1111/1462-2920.70354 (PMC13278821; doi:10.1111/1462-2920.70354)
Supplement: Supplementary file 1 — Figure S1: Targeted deletion of FgJHD2 in F. graminearum. (A) Through homologous recombination, the coding sequence of Fgjhd2 was replaced with the hygromycin resistance gene (hyg). Using double‐ligation PCR, the upstream and downstream homologous arms (HY and YG) of Fgjhd2 were ligated with the hyg fragment to construct a knockout fragment for fungal transformation. (B) PCR identification results for the knockout mutant strains. Validation was performed using specific primers located on the outer side of the homologous arms to detect gene replacement. The successfully obtained knockout mutant strains (Fgjhd2‐1 and Fgjhd2‐2) exhibited expected amplification bands, while no hyg fragment signal was detected in the wild‐type strain (PH‐1). Figure S2: FgJhd2 no affects the conidial morphology or production and of F. graminearum. (A) Conidium length of WT, Fgjhd2 mutant strain and complementary strain. (B) Conidium production of WT, Fgjhd2 mutant strain and complementary strain. Bars represent the standard deviation. (C) Virulence assays on flowing wheat heads with conidia of the PH‐1, Fgjhd2 and Fgjhd2‐C strains. Photographs were taken at 14 days after inoculation (dpi). (D) Disease index counted after 14 dpi. Data was analysed with one‐way ANOVA, *p < 0.05, ns, not significant. Figure S3: FgJhd2 affect the growth and stress response of F. graminearum. (A) Wild‐type (PH‐1) and Fgjhd2 mutant strains were cultured on potato dextrose agar (PDA) plates supplemented with 0.02% SDS, 0.05% Congo red, 1.5 M NaCl, 20 mM H2O2 at 25°C for 3 days. (B) Colony diameter of the strains on PDA medium. Data was analysed with two‐way ANOVA, *p < 0.05, ns, not significant. Figure S4: Genome‐wide distribution of ChIP‐seq signals in PH‐1 and the Fgjhd2 mutant. Figure S5: Sequence alignment analysis revealing that Fgmpf2 shares high similarity with the meiotic Pumilio family RNA‐binding protein SpMpf2 from S. pombe. Figure S6: Analysis of different mutant phenotypes. (A) Colony morphology of [file EMI-28-e70354-s001.docx]

## Figure S1


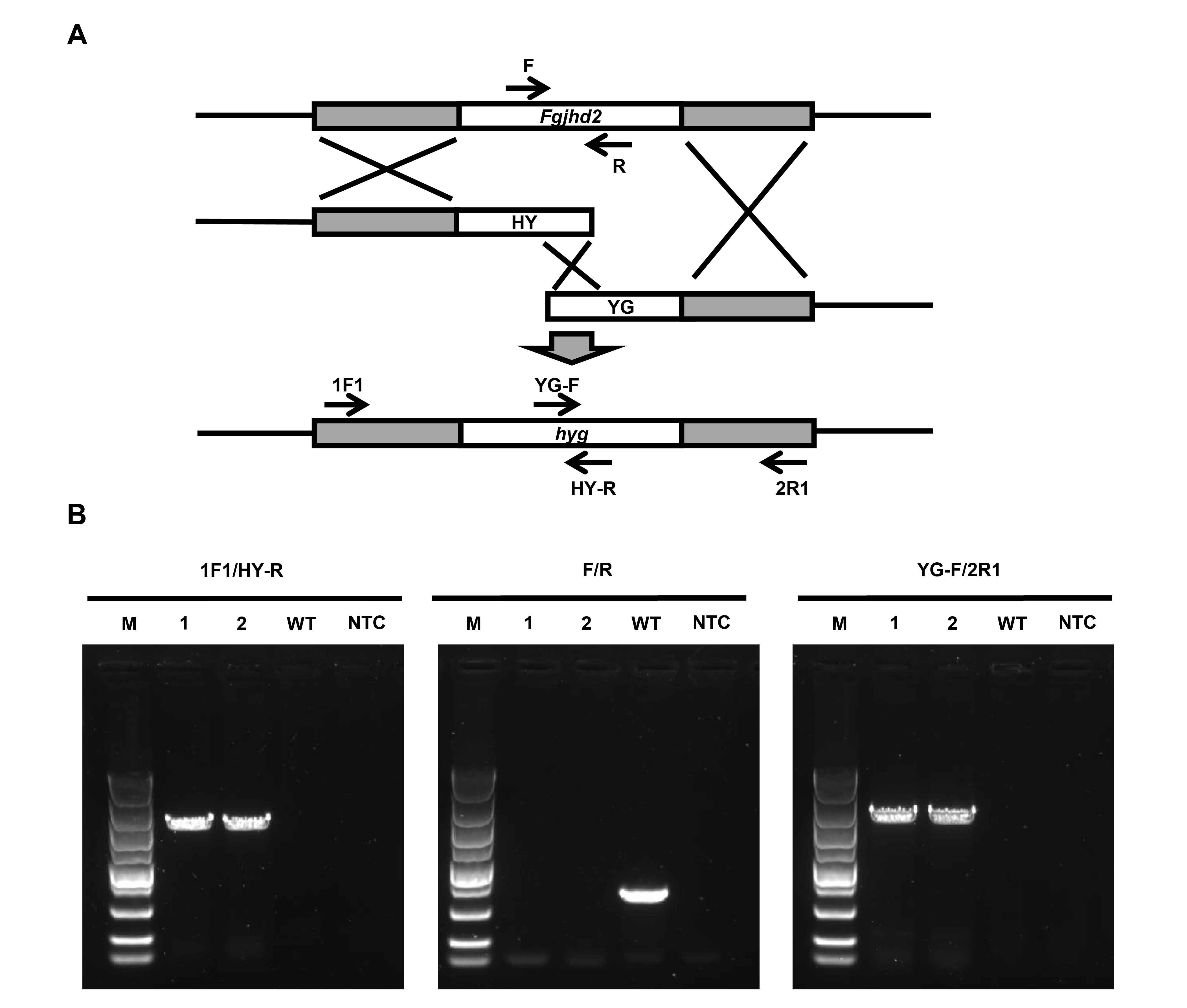


**Fig.S1** Targeted deletion of *FgJHD2* in *F. graminearum*. (A) Through homologous recombination, the coding sequence of *Fgjhd2* was replaced with the hygromycin resistance gene (*hyg*). Using double-ligation PCR, the upstream and downstream homologous arms (HY and YG) of *Fgjhd2* were ligated with the *hyg* fragment to construct a knockout fragment for fungal transformation. (B) PCR identification results for the knockout mutant strains. Validation was performed using specific primers located on the outer side of the homologous arms to detect gene replacement. The successfully obtained knockout mutant strains (*Fgjhd2-1* and *Fgjhd2-2*) exhibited expected amplification bands, while no *hyg* fragment signal was detected in the wild-type strain (PH-1).

## Figure S2


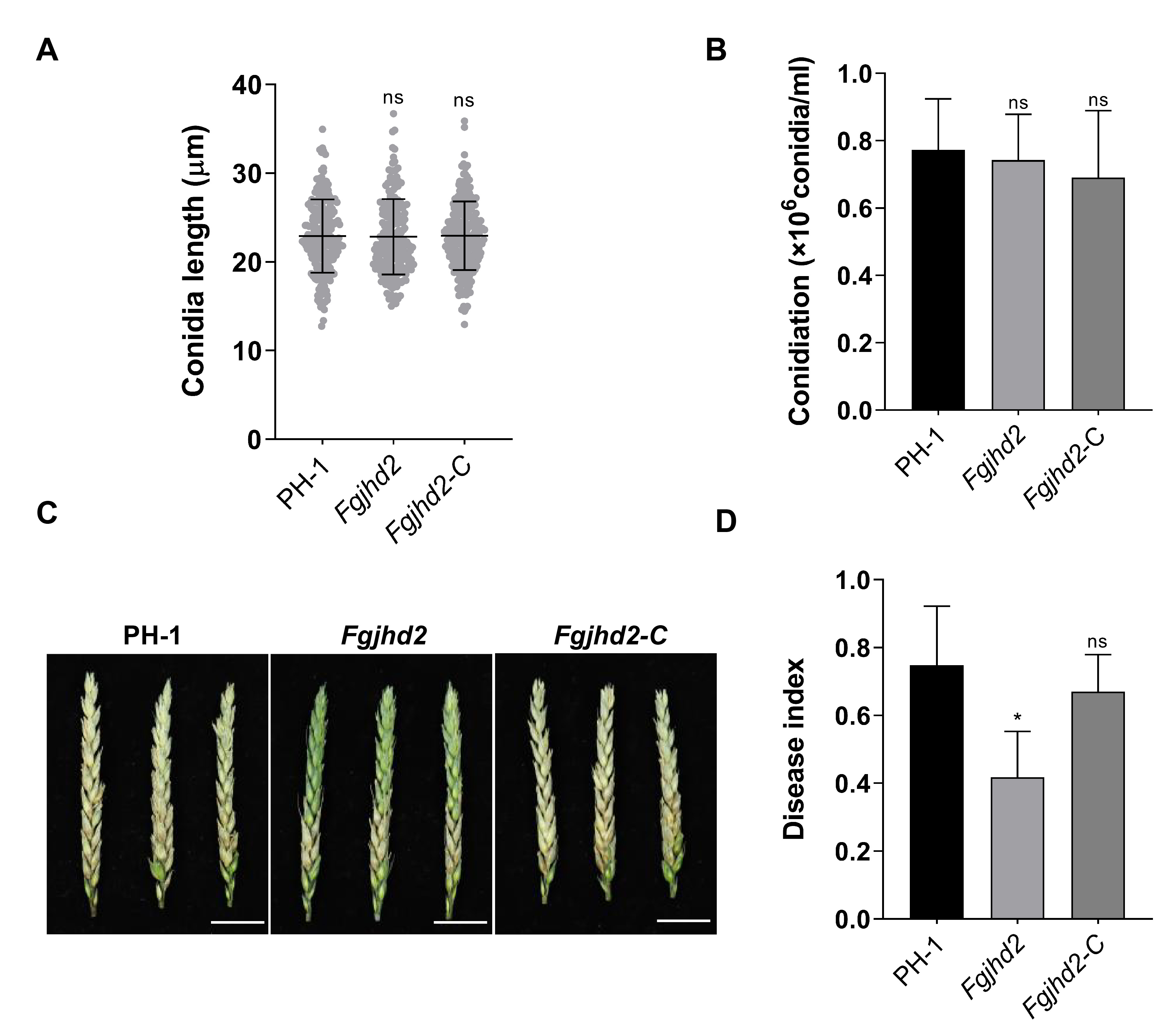


**Fig.S2** FgJhd2 no affects the conidial morphology or production and of *F. graminearum.* (A) Conidium length of WT, *Fgjhd2* mutant strain and complementary strain. (B) Conidium production of WT, *Fgjhd2* mutant strain and complementary strain. Bars represent the standard deviation. (C) Virulence assays on flowing wheat heads with conidia of the PH-1, *Fgjhd2*, and *Fgjhd2-*C strains. Photographs were taken at 14 days after inoculation (dpi). (D) Disease index counted after 14 dpi. Data was analyzed with one-way ANOVA, *, *P* < 0.05, ns, not significant.

## Figure S3


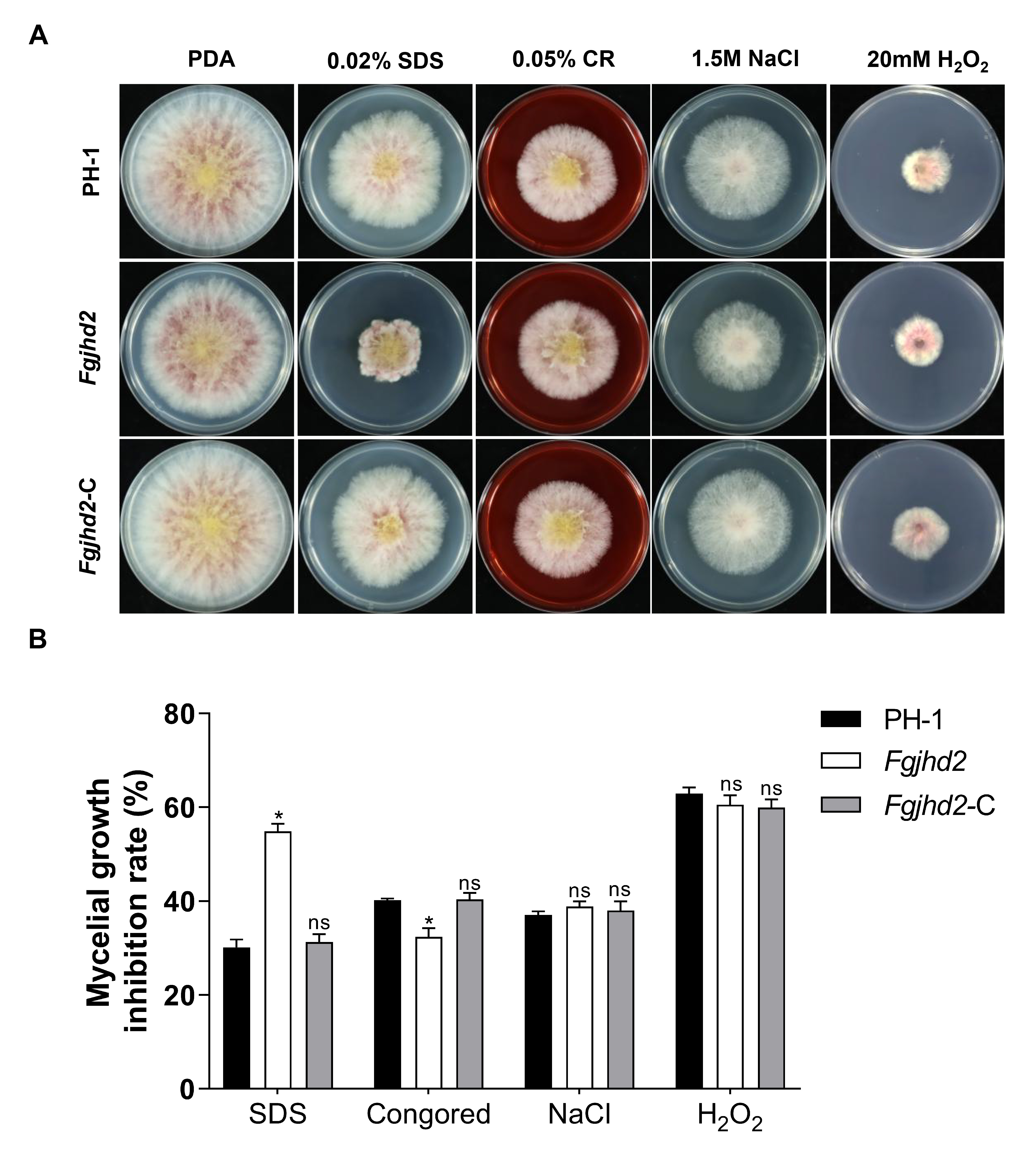


**Fig.S3** FgJhd2 affect the growth and stress response of *F. graminearum.* (A) Wild-type (PH-1) and *Fgjhd2* mutant strains were cultured on potato dextrose agar (PDA) plates supplemented with 0.02％ SDS, 0.05% Congo red, 1.5 M NaCl, 20 mM H_2_O_2_ at 25°C for 3 days. (B)Colony diameter of the strains on PDA medium. Data was analyzed with two-way ANOVA, *, *P* < 0.05, ns, not significant.

## Figure S4


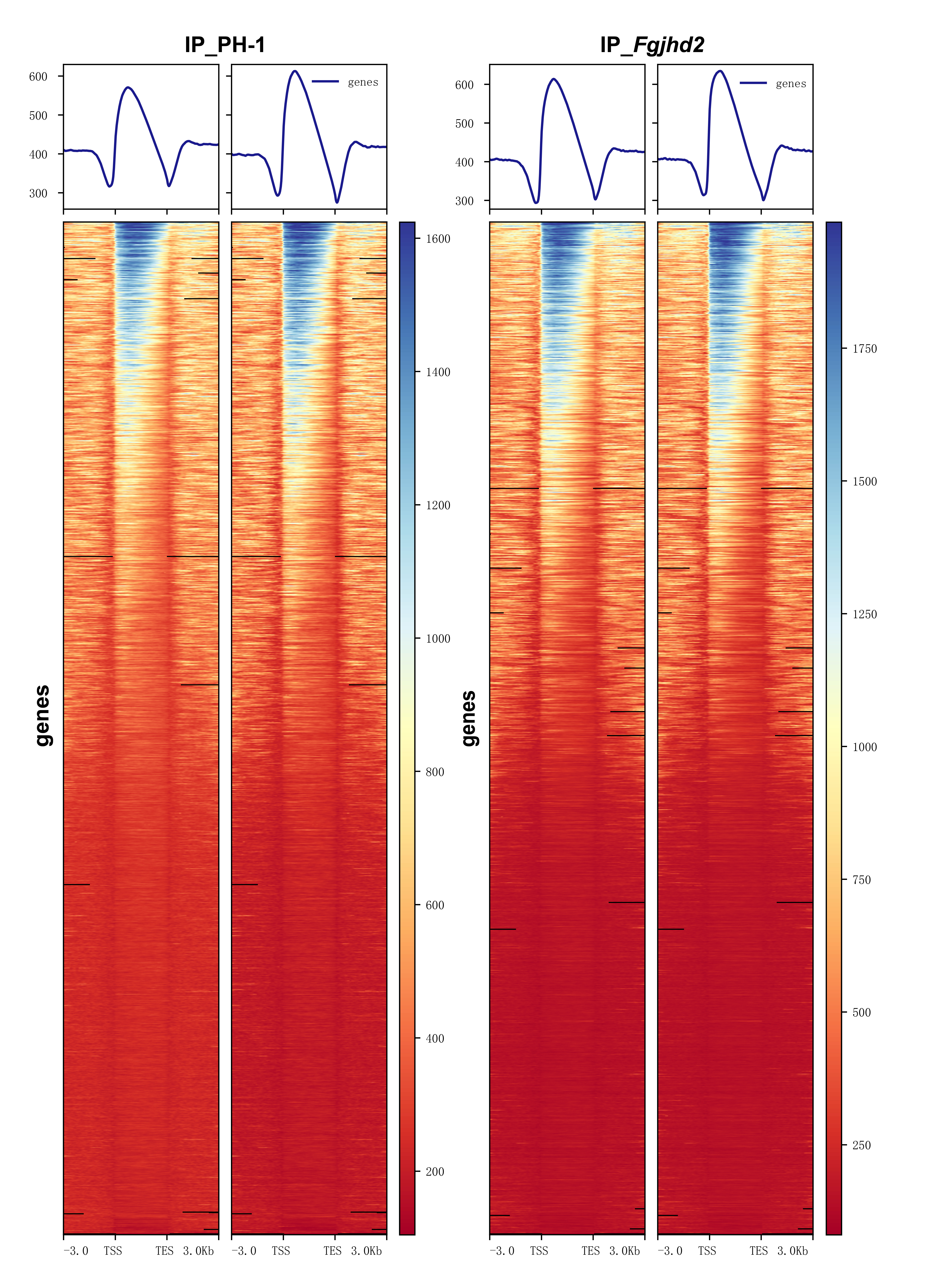


**Fig.S4** Genome-wide distribution of ChIP-seq signals in PH-1 and the *Fgjhd2* mutant..

## Figure S5


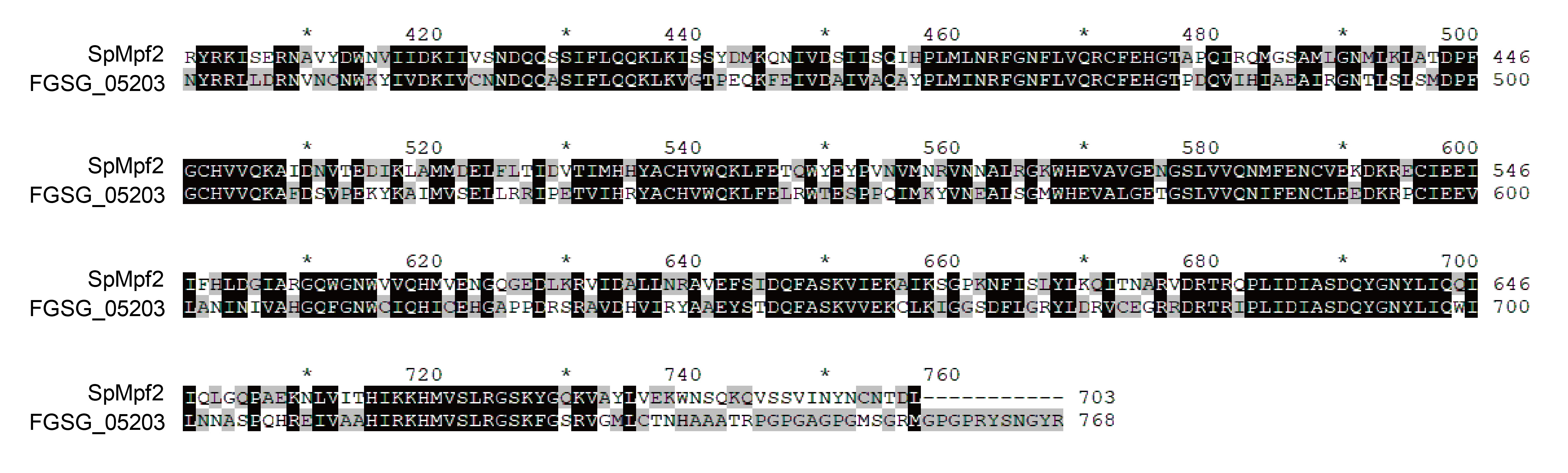


**Fig.S5** Sequence alignment analysis revealing that Fgmpf2 shares high similarity with the meiotic Pumilio family RNA-binding protein SpMpf2 from S. pombe.

## Figure S6


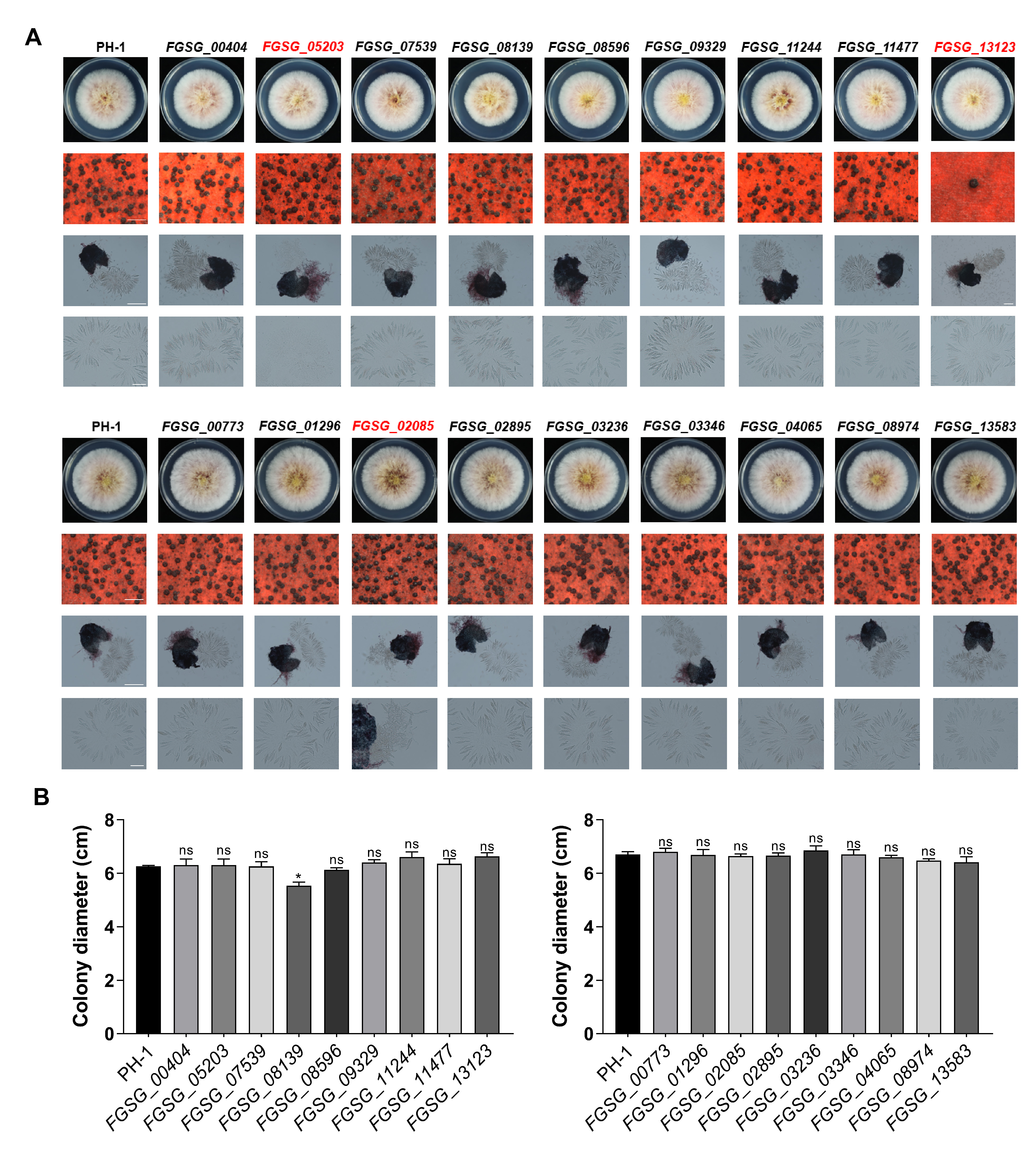


**Fig.S6** Analysis of different mutant phenotypes. (A) Colony morphology of 18 gene deletion mutants selected from transcriptomic clustering after 3 days of incubation on PDA, and perithecial, ascus, and ascospore development observed on carrot agar at 7 dpf. Bar = 200 µm (Upper), bar = 20 µm (Bottom). (B) Comparison of colony diameters of the PH-1 and 18 gene deletion mutants strains on PDA after 3days. Data was analyzed with one-way ANOVA, *, *P* < 0.05, ns, not significant.

## Figure S7


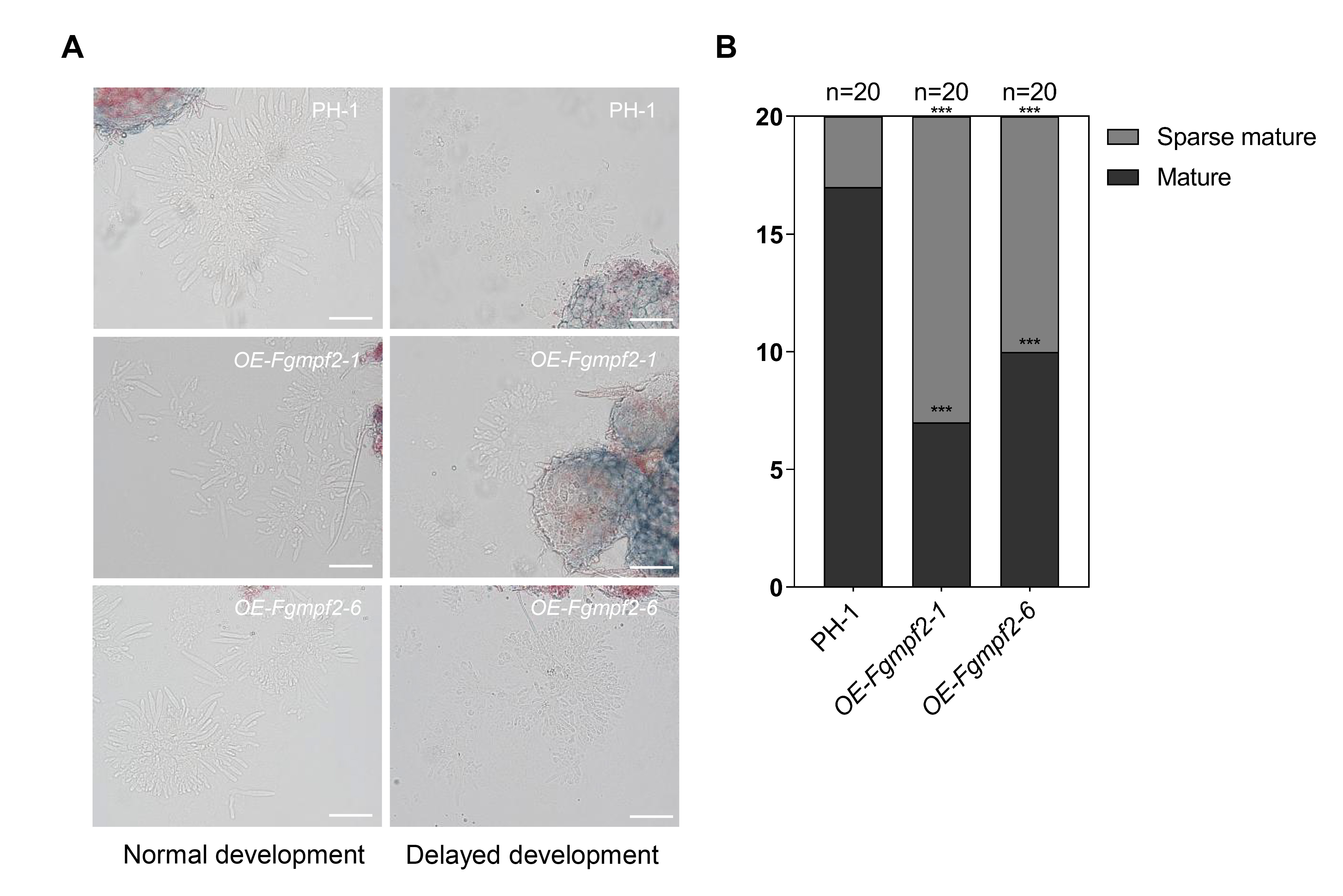


**Fig.S7** Overexpression of *Fgmpf2* affects perithecial maturation and ascospore development in *F. graminearum*. (A) Microscopic observation of asci and ascospores in PH-1 and *OE-Fgmpf2* transformants 5 days postfertilization. bar = 20 µm. (B) Statistical analysis of morphology in ascomata and ascospores of PH-1 and *Fgmpf2* overexpressing strains. Data was analyzed with one-way ANOVA, *, *P* < 0.05, ***, *P* < 0.001.

## Figure S8


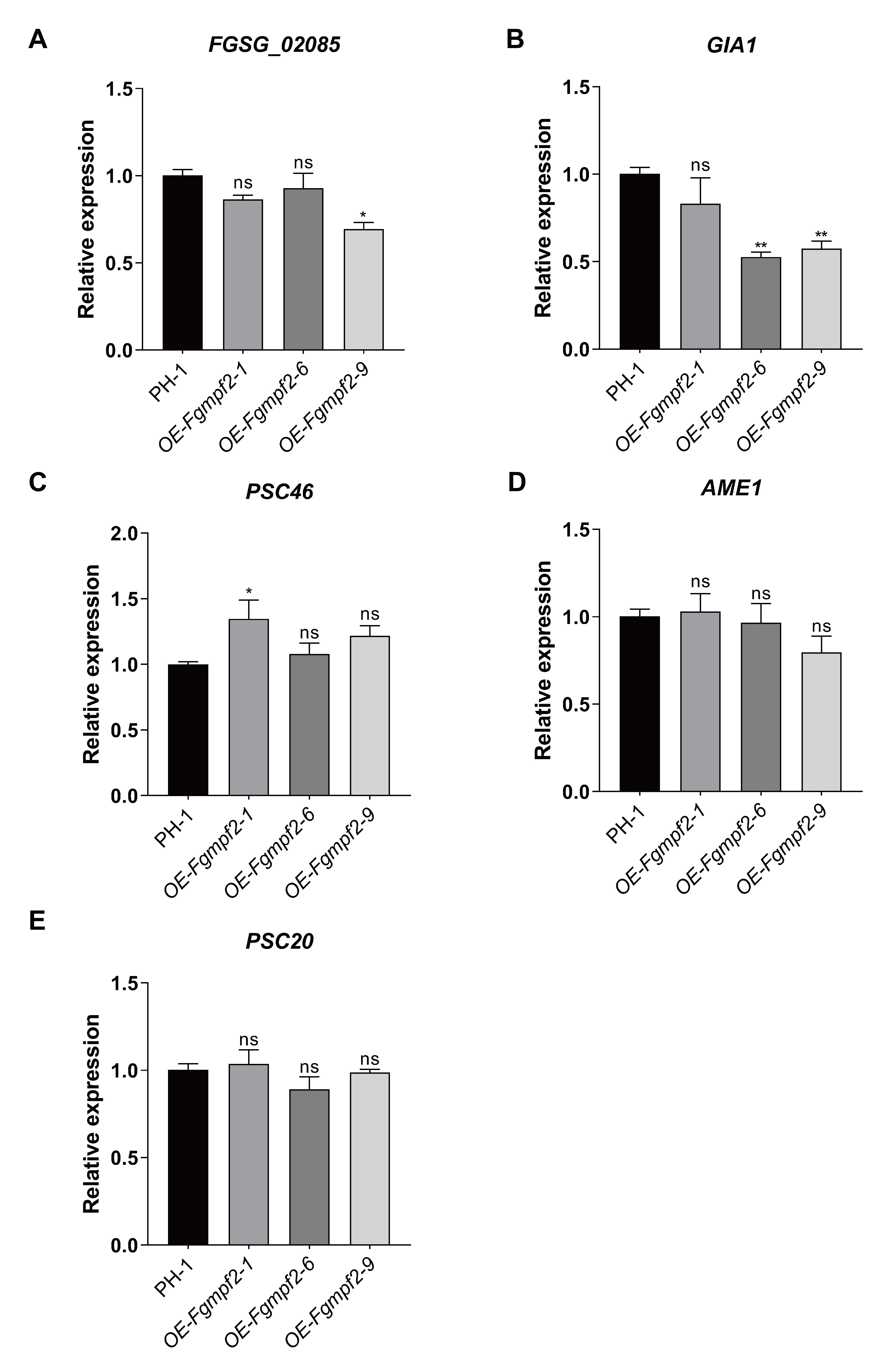


**Fig.S8** Expression analysis of *FGSG_02085*, *GIA1*, *PSC46, AME1* and *PSC20* in the *OE-Fgmpf2* strain. Data was analyzed with one-way ANOVA, *, *P* < 0.05, ns, not significant.
